# Cell-imaging studies of highly substituted oxazole derivatives as organelle targeting fluorophores (OTFPs)

**DOI:** 10.1038/s41598-022-20112-y

**Published:** 2022-10-03

**Authors:** Saswati Adhikary, Kaustuv Mukherjee, Biswadip Banerji

**Affiliations:** 1 Organic and Medicinal Chemistry Division, Indian Institute of Chemical Biology (CSIR-IICB), 4 Raja S. C. Mullick Road, Kolkata, 700032 India; 2 Academy of Scientific and Innovative Research (AcSIR), Ghaziabad, 201002 India; 3 Cancer Biology & Inflammatory Disorder Division, Indian Institute of Chemical Biology (CSIR-IICB), 4 Raja S. C. Mullick Road, Kolkata, 700032 India

**Keywords:** Chemical biology, Chemistry

## Abstract

The field of biomedical science has progressed enormously in the past decade. With the advent of newer diagnostic tools for imaging and detection, identification of root cause of a disease is now remarkably accurate and specific. Small organic fluorophores in these connections are in great demand currently for cellular organelle sensing and detecting, due to their non-invasiveness, excellent accuracy and bio-sensitivity. Small molecule fluorescence probes offer most potent area for biological sensing with diagnostic imaging ability. These organelle targetable fluorescent probes are produced through synthetic manipulations to get the desired, decent fluorescence properties. When a suitable organelle specific functional group is installed within these highly fluorescent scaffolds, then these molecules turn out to be as lysotracker, mitotracker and cytoplasm-stainer in mammalian cells with high efficiencies (high Pearson co-efficient factors). The present work demonstrated an environmentally benign (green) one-pot, sp^3^ C–H functionalization of highly substituted oxazole derivatives with excellent photophysical properties. These molecules were further modified by installing organelle specific targetable groups (sensors/detectors) which selectively localize in specific intra-cellular organelles.

## Introduction

**Figure 1:**
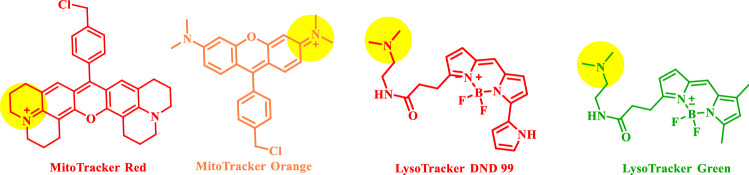
Some examples of small organic fluorophores.

Sub-cellular organelles are extremely significant part of cells which maintain the basic requirements of life through versatile biochemical reactions and their information regarding structure, morphology can be gathered by organelle targetable fluorescence probes (OTFPs) (Fig. 1)^1^. These different biochemical reactions take place simultaneously within several organelles, which are needed for cell survival. Also, for proper cellular functioning, coordination among different organelles is essential. For all these biochemical events, the role of mitochondria, lysosomes and cytoplasm are essential for maintaining cellular homeostasis^2–4^. Mitochondria, the most dynamic and vital sub-cellular organelle, is critical for many life-sustaining biochemical activities including cellular respiration, energy currency-adenosine triphosphate (ATP) production, cellular signalling, reactive oxygen species (ROS) generation, cell cycle, cell growth, signalling, apoptosis etc. cellular actions are maintained by all these activities^2^. Healthy mitochondria population is preserved by removing damaged mitochondria called mitophagy^5^. With the onset of different physiological and disease states, mitochondrial number, position, dynamics and morphology changes occur. Thus, visualizing and tracking mitochondria are very significant to understand different physiological and pathological conditions for diagnosis and treatment^6^. Here, the role of fluorescent probes is extremely critical.

**Figure 2:**
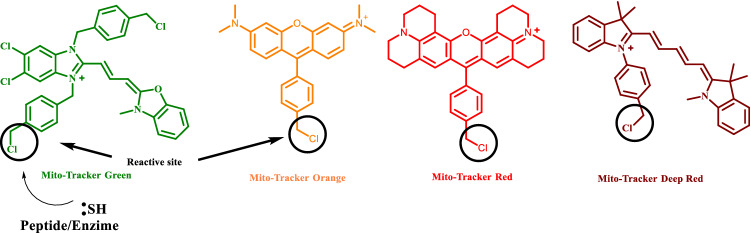
Some examples of reaction-based mitochondria staining probes.

Mitochondrial fluorescence probes are divided into two categories: reaction-based cationic probes and electrostatic interaction based lipophilic cationic probes^6^. Due to mitochondrial negatively charged membrane potential, lipophilic cationic probes can be easily located in it due to electrostatic interaction. On the other hand, reaction-based mito-tracker probes contain a reactive benzyl chloride site that easily participate in chemical reaction with thiol group of peptides and proteins inside the mitochondria, causing high cytotoxicity (Fig. 2)^7^. This issue leads to a high demand for development of a reaction-free strategy based, bio-compatible new generation bio-probes, which is extremely challenging.

Lysosome is another dynamic organelle that maintains cellular homeostasis by degradation of different macromolecules including peptides, nucleic acids, carbohydrates, lipid etc. by several acid hydrolases (pH ≤ 5). Lysosomes participate in intracellular signalling, recycling damaged organelles, repairing plasma membrane, inactivating extracellular antigens, autophagy etc. Also, it is involved in antigen processing, display, and antibody production against pathogens^3^. Lysosomal dysfunctions result in the onset of several diseases^8^. To better understand the status of lysosomes, its visualisation and tracking are highly important^1^. Though different acidotropic Lyso-tracker probes are available, most of these are suffering from biocompatibility issues^9^. Thus, to overcome the drawback of the mentioned issues and specifically stain lysosome, development of new more potent biocompatible probes are in high demand.

The cytoplasm is the gel-like fluid inside the cell. It is in charge of holding cell components and protecting them from damage. It stores the molecules needed for cellular processes and is also responsible for shaping the cell. Protein synthesis, cell division, metabolic reactions, cellular signals, cellular shape etc. many life-sustaining processes are going on within the cytoplasm^4^. Thus, visualization of cytoplasm is essential to gather the information regarding cellular status.

The non-invasiveness, small size, easy chemical modification, excellent reproducibility, biocompatibility and accuracy of small molecule OTFPs allow real-time biological sensing along with diagnostic imaging ability^7^. Scientists prefer OTFPs as these can potentially label specific sub-cellular organelles through which important information can be gathered that helps to understand different cellular functions and their ambiguities (Fig. 1). Although inorganic metal nanoparticles are blessed with favourable advantages, still, their low biodegradability and toxicity poses serious problems for clinical trials. To cope with these problems and to make them more potent for treatment, small organic molecule based sub-cellular OTFPs with potential towards synthetic flexibility are being developed^10^. Luminescent small organic fluorescent probes thus become a viable alternatives to organic fluorophores for sub-cellular targeting and imaging applications due to the following advantages: (i) synthesis of small organic molecule with one-pot green synthetic protocols; (ii) tunable excitation and emission maxima by modification of the ancillary ligands; (iii) large Stokes shift that facilitate the separation of excitation and emission wavelengths; (iv) good water solubility for mitotracker dye and biocompatibility.

Recently our group reported imidazole based new class of lysosome tracking fluorophore where a lysosome directing group was anchored based on donor-π-acceptor (D-π-A) model^11^. After successful evaluation of these molecules as lysosome tracking agent, we next tried to broaden the scope of our work which can be tuned accordingly.

**Figure 3:**
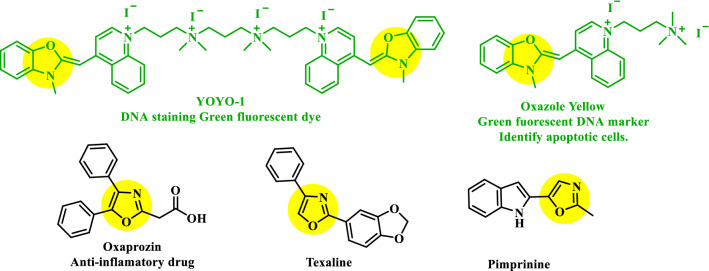
Some famous oxazole containing compounds and natural products.

**Figure 4:**
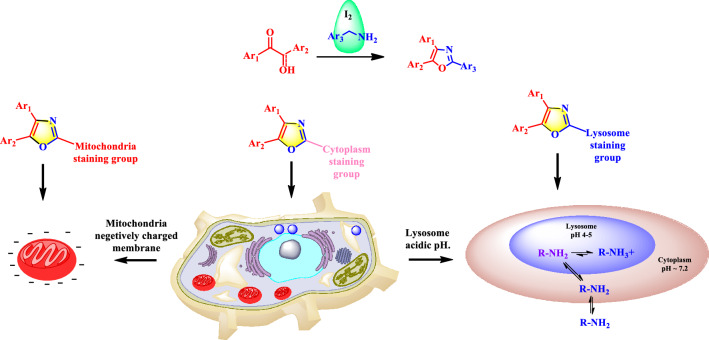
Our design strategy: to produce different small organic bio-fluorophores (mitotracker, lysotracker and cytoplasm stainer) by simply manipulating Ar3- groups in the parent oxazole scaffold.

Oxazole is a potent scaffold found in numerous biologically active compounds, and natural products (Fig. 3). This potent scaffold can act as either electron donors or acceptors that facilitate D-π-A interaction. This extends π-conjugation and exhibited excellent fluorescence responses^[17]^. Here diversified oxazole scaffolds were synthesized simply by changing the starting materials via environmentally benign, iodine catalyzed open-air C(sp^3^)-H oxidative cyclization reaction^[17]^. The flexible design strategies (Fig. 4) provided the scopes for tuning the designed small organic fluorescent probes accordingly that potentially and selectively can target the sub cellular organelle with minimal cytotoxicity and high biocompatibility^7,11^. These fluorophores can now be tested to detect cellular organelles and biomolecules in biological research. This study therefore unlocked oxazoles as a new class of luminophores with immense potential to be explored in the bio-imaging field.

## Results and discussion

### Molecular design

The small organic molecule based sub-cellular organelle targetable fluorophores were designed including (a) fluorophore, (b) sub-cellular organelle targetable group and (c) D-π-A framework. Highly substituted oxazole scaffolds exhibiting good fluorescence responses^11^, that can be tuned towards bio-fluorophore simply by conjugating suitable sub-cellular organelle specific group. Moreover, incorporation of D-π-A framework, improve the fluorescence efficacy.

### Synthesis and characterization

**Scheme 1:**
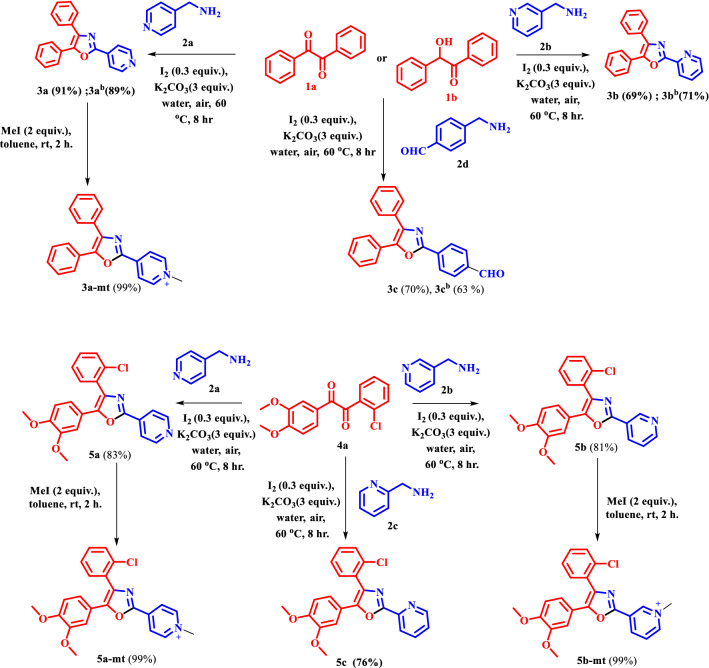
Synthesis of different organelle targetable fluorophores.

A brief description of the synthetic procedure is outlined in Scheme 1. The target bio-fluorophores **3a, 3b** and **3c** were synthesized by taking together benzil (1 equiv.)/benzoin (1 equiv.) with amine (1 equiv.) in presence of iodine (30 mol%) and potassium carbonate (3 equiv.) in water and stirred at 60 °C for 8 h. By using the same reaction condition, **5a, 5b** and **5c** were synthesized from di-ketone **4a** and amine **2a, 2b** and **2c** respectively^11^. **3a, 5a** and **5b** then methylated (MeI/toluene, rt) that provide **3a-mt, 5a-mt** and **5b-mt** respectively (Scheme 1). All the molecules were characterized by ^1^H, ^13^C-NMR and HRMS spectroscopic studies (see the characterization section in the ESI).

**Figure 5:**
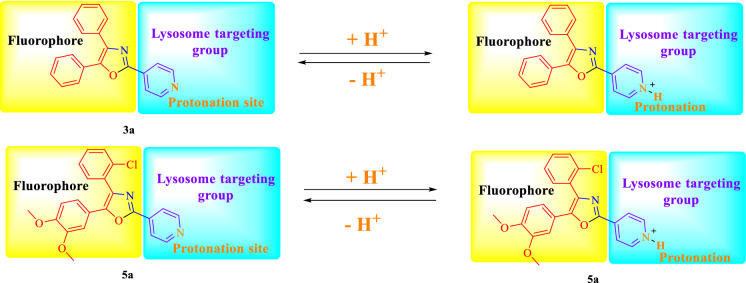
pH response behaviour for lysosome targetable group^13^.

### Photophysical properties

#### Solvatochromism and intramolecular charge transfer of oxazole derivatives

**Figure 6:**
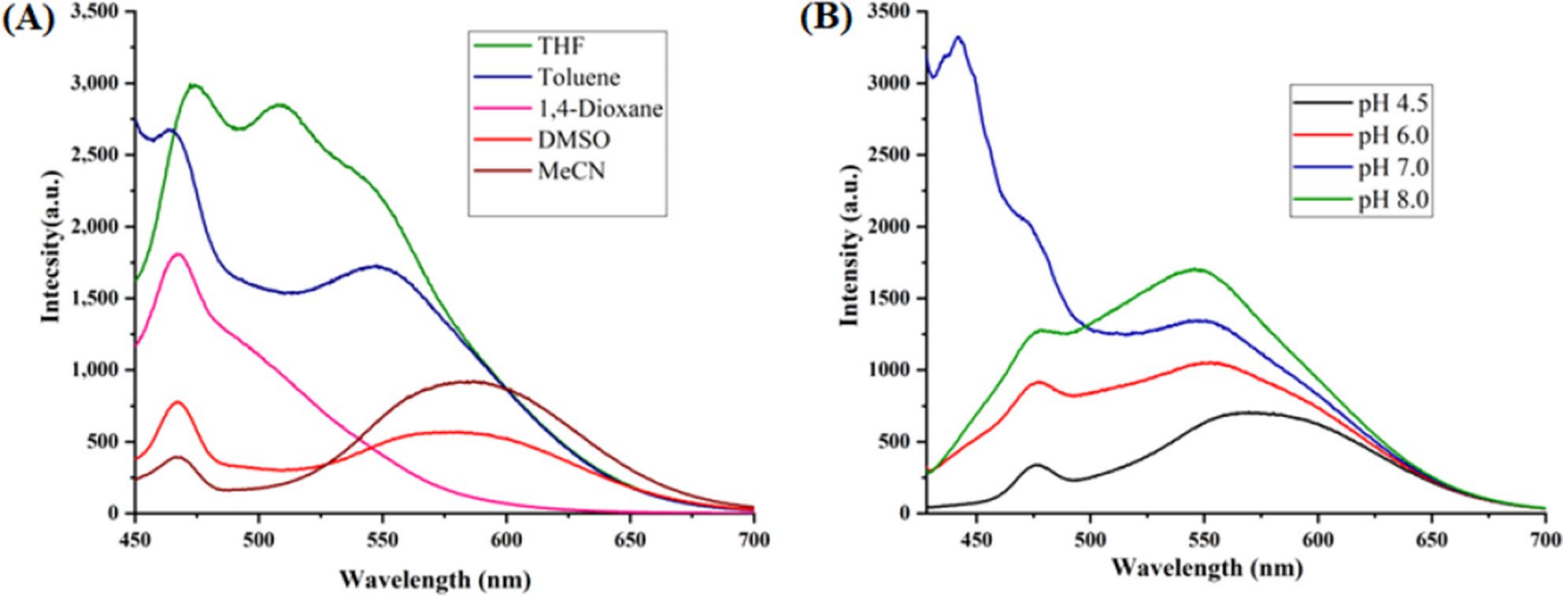
(**A**) Fluorescence spectra (concentration: 500 nM) of **5a-mt** in different solvent, (**B**) 10 μM solution of **5a-mt** compound in different pH-buffer solution.

**Figure 7:**
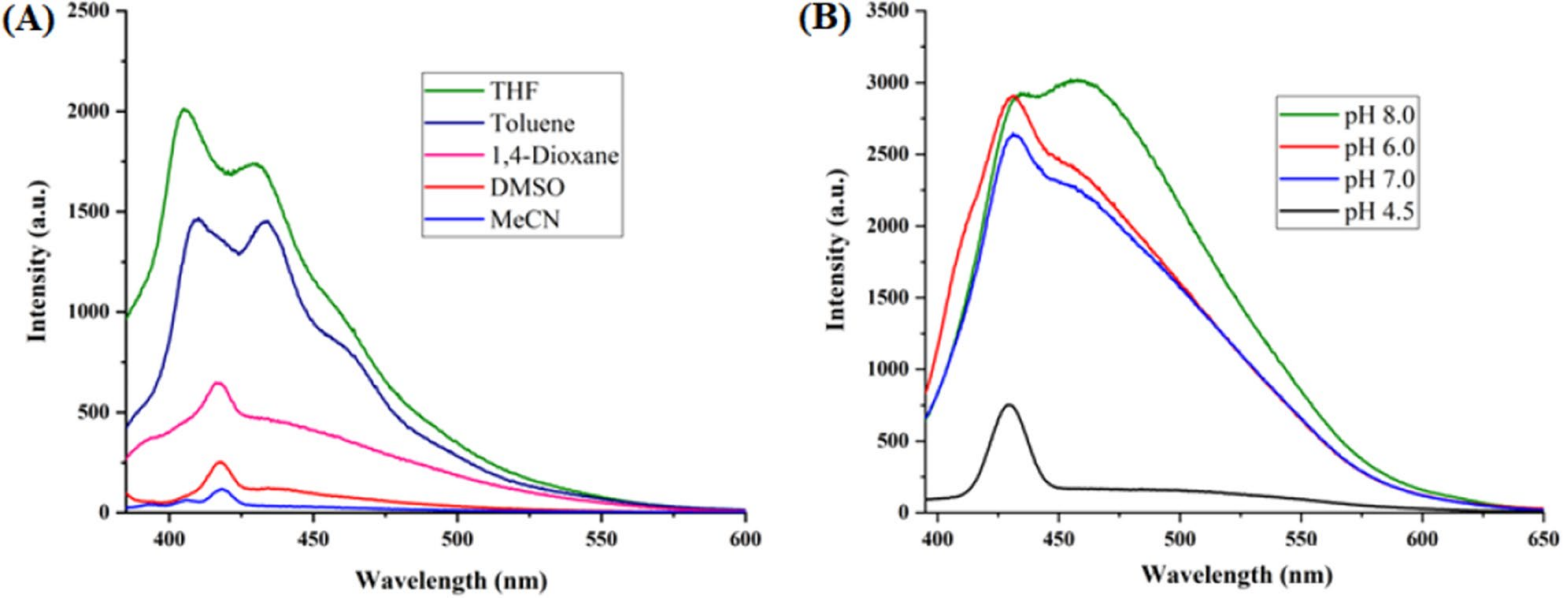
(**A**) Fluorescence spectra (concentration: 500 nM) of **5b-mt** in different solvent, (**B**) 10 μM solution of **5b-mt** compound in different pH-buffer solution.

**Figure 8:**
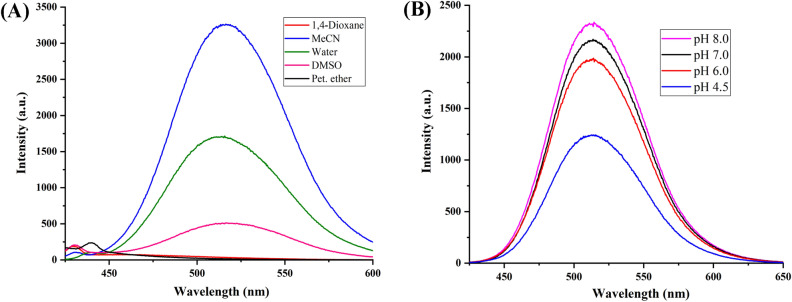
(**A**) Fluorescence spectra (concentration: 500 nM) of **3a-mt** in different solvent, (**B**) 10 μM solution of **3a-mt** compound in different pH-buffer solution.

**Figure 9:**
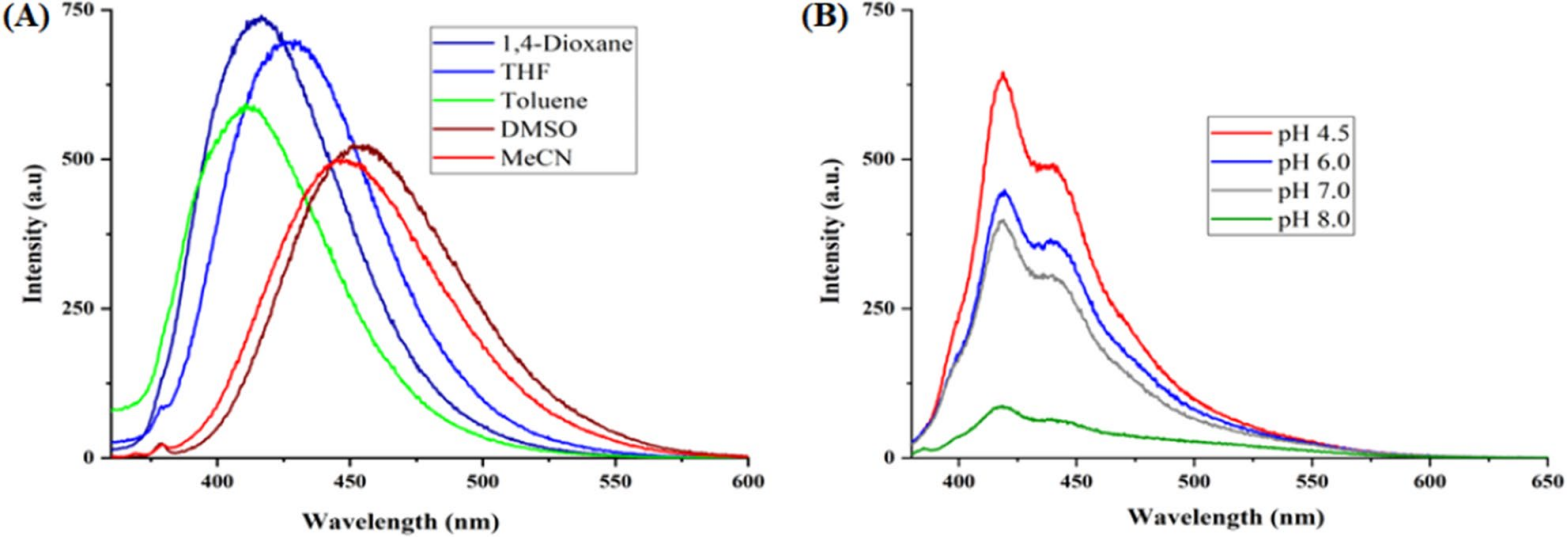
(**A**) Fluorescence spectra (concentration: 500 nM) of **5a** in different solvent, (**B**) 10 μM solution of **5a** compound in different pH-buffer solution.

**Figure 10:**
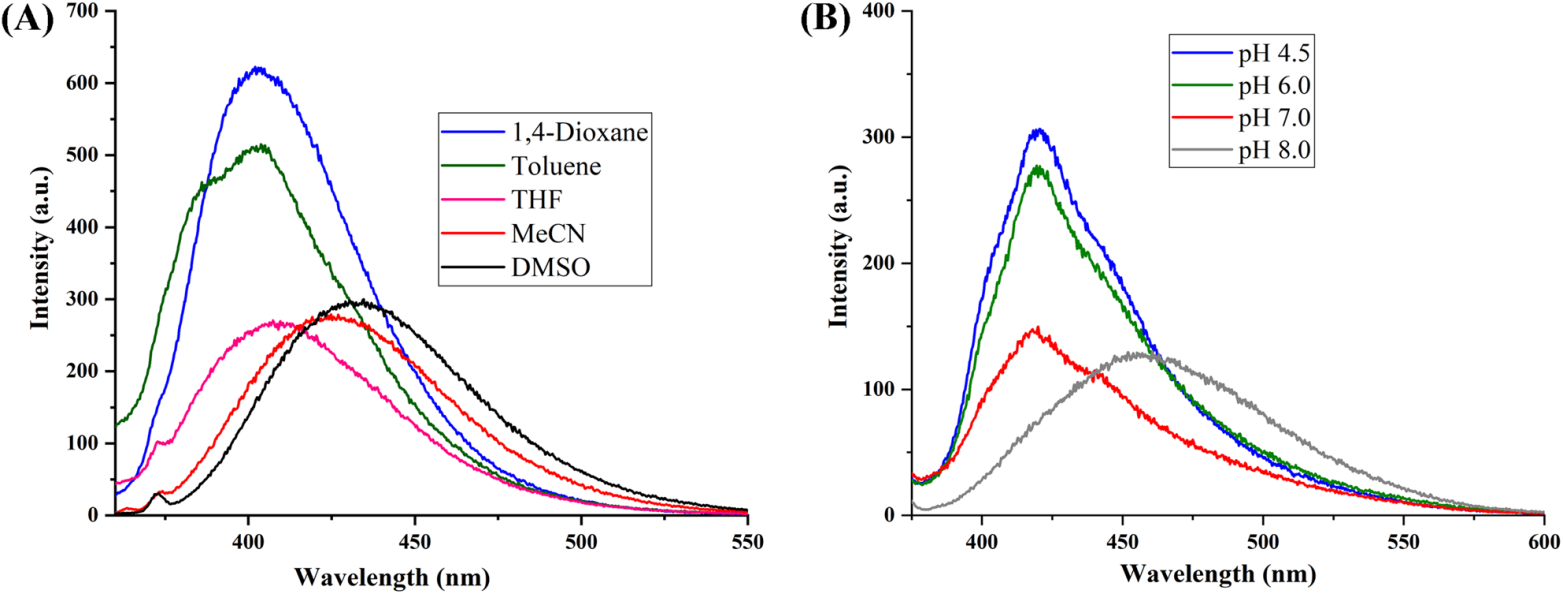
(**A**) Fluorescence spectra (concentration: 500 nM) of **5b** in different solvent, (**B**) 10 μM solution of **5b**compound in different pH-buffer solution.

**Figure 11:**
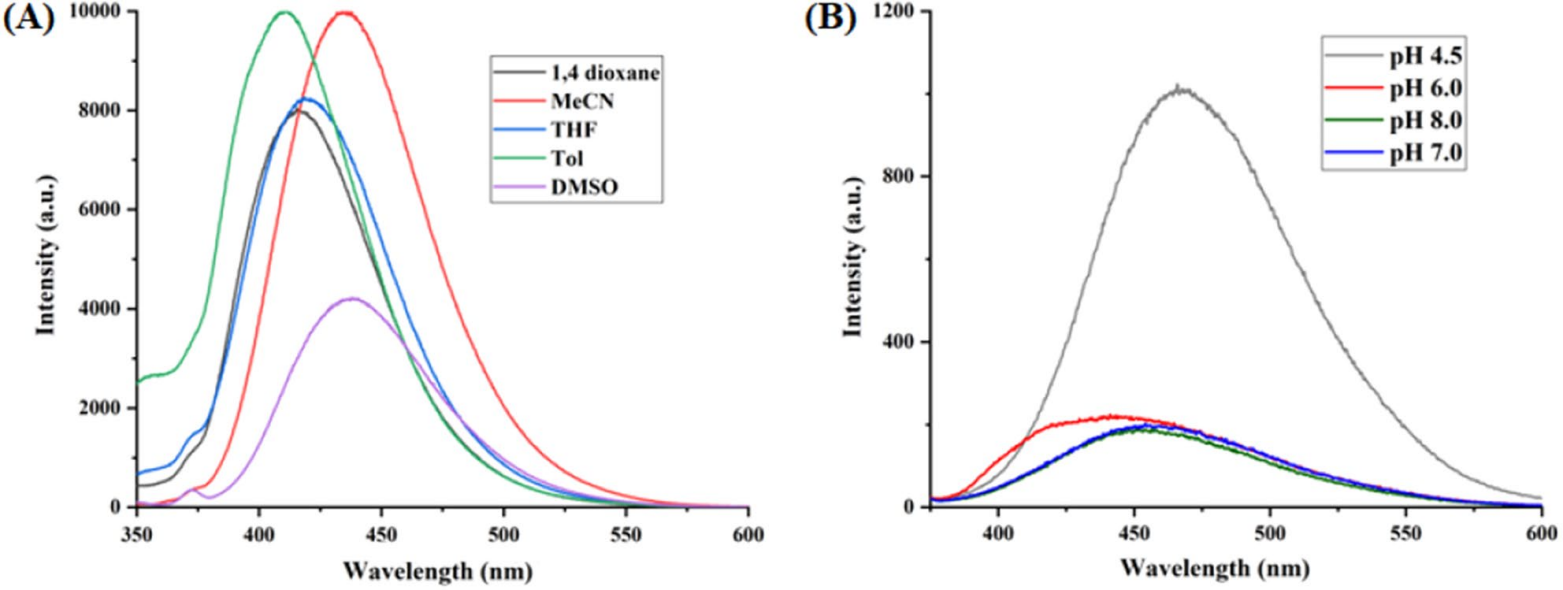
(**A**) Fluorescence spectra (concentration: 500 nM) of **5c** in different solvent, (**B**) 10 μM solution of **5c**compound in different pH-buffer solution.

**Figure 12:**
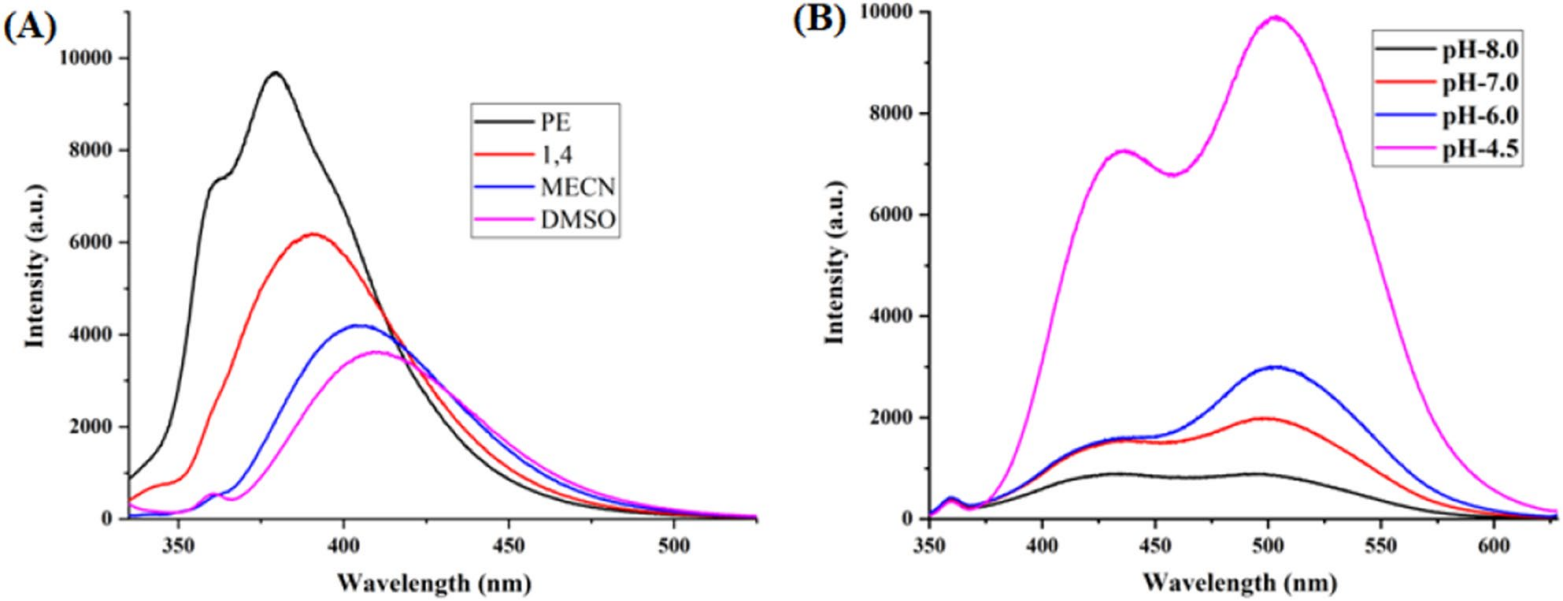
(**A**) Fluorescence spectra (concentration: 500 nm) of **3a** in different solvent, (**B**) Fluorescence spectra of 10 μM solution of **3a** compound in different pH-buffer solution.

**Figure 13:**
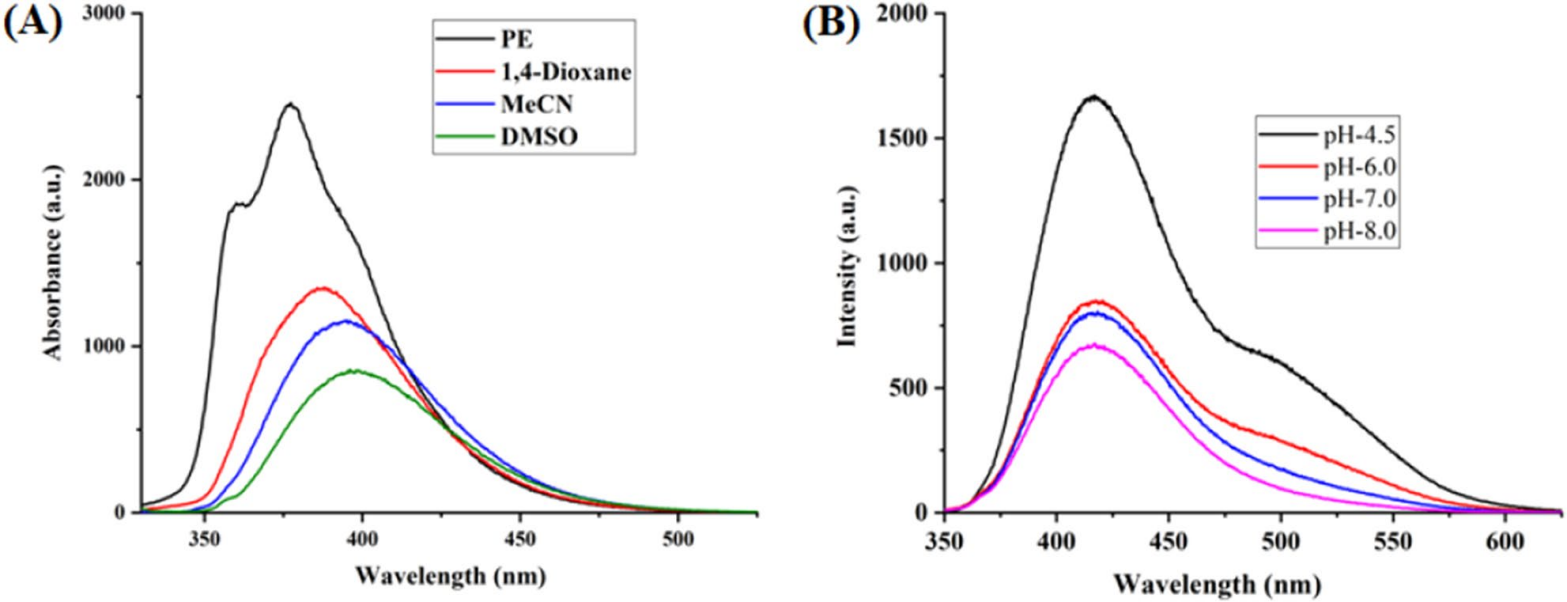
(**A**) Fluorescence spectra (concentration: 500 nm) of **3b** in different organic solvent, (**B**) Fluorescence spectra of 10 μM solution of **3b**compound in different pH-buffer solution.

**Figure 14:**
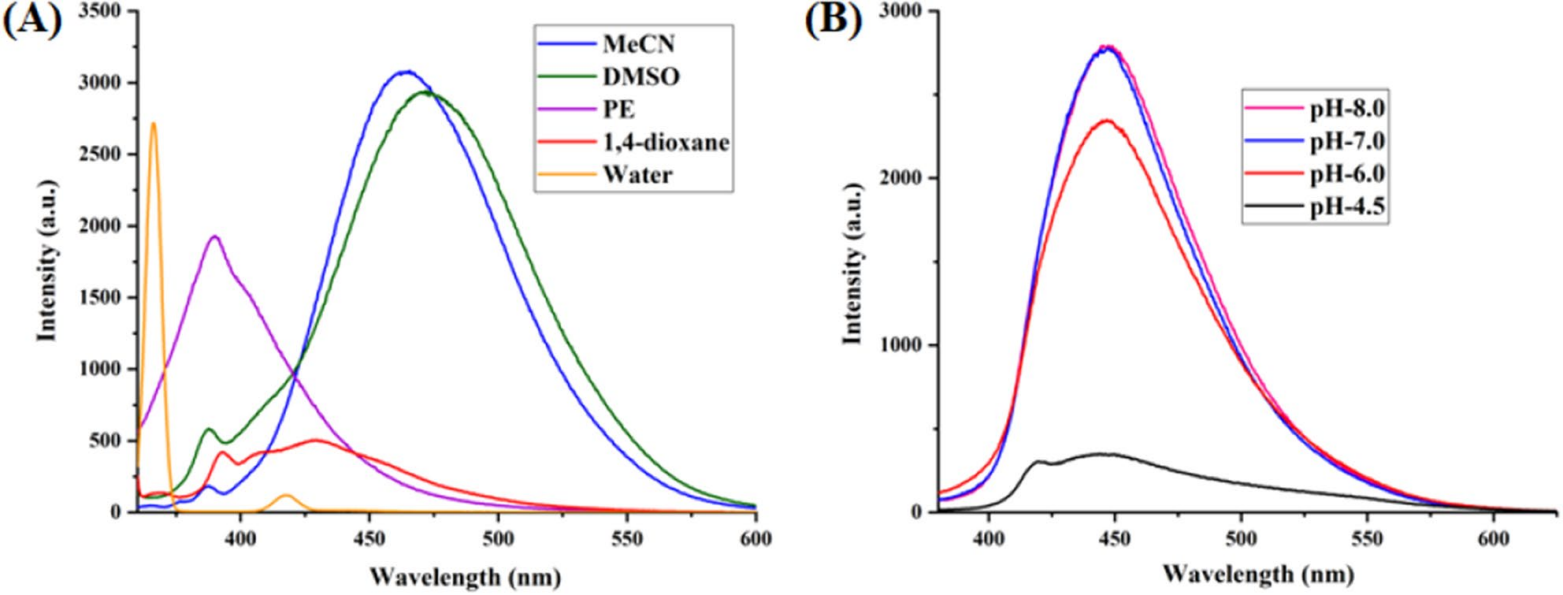
(**A**) Fluorescence spectra (concentration: 500 nm) of **3c** in different solvent, (**B**) Fluorescence spectra of 10 μM solution of **3b** in different pH-buffer solution.

The optical properties of highly substituted oxazole derivatives were then scrutinized using different solvents and pH (Fig. 5). Oxazole scaffold may serve as either an electron donating or withdrawing group, depending on the substitution to it, provided strong D-π-A structures. The solvatochromic effect of these oxazole derivatives were studied in various solvents with different polarities. The emission spectrum shifted towards longer-wavelength region accompanying with a decrease in the emission intensity (Figs. 6A, 7A, 8A, 9A, 10A, 11A, 12A, 13A and 14A). This result demonstrates of an intramolecular charge transfer (ICT) effect caused by the interaction between the electron-donating unit (phenyl/methoxyphenyl/chlorophenyl) and the electron-accepting pyridinium moiety^14^. For example, keeping electron-withdrawing 4-methyl pyridinium group fixed, with substitution of -Ph group with more electron donating dimethoxy phenyl and chlorobenzyl group, showed red shifting in emission spectroscopy. The bathochromic shift indicated the increasing electron donating ability (phenyl < methoxyphenyl group), that will strengthen D-π-A effect^14^. For example, **5a-mt** and **3a-mt** exhibited bathochromic shift relative to **5a** and **3a** respectively. Similarly, **5a** and **5c** also showed the similar trend relative to **3a**, and **3b** respectively.

### pH dependent emission spectroscopy

Lysosomes have V-ATPases that pumps proton and maintain the luminal pH environment at a range of pH 4.6–5.0. This lysosomal acidity is vital for maintaining optimal activities for different hydrolases^15^. Thus the pH sensitivity of these oxazole derivatives was then investigated. A significantly enhanced fluorescence signals were appeared for **5a, 5b, 5c, 3a** and **3b** with decrease pH from 8.0 to 4.5, demonstrating that these molecules are acid-responsive (Fig. 6B, 7B, 8B, 9B, 10B, 11B, 12B, 13B and 14B). Due to fluorescent intensity enhancement of the mentioned molecules at the low pH, these compounds can be readily visualised in lysosomal low acidic environment. The enhancement of fluorescence in low acidic condition is due to intramolecular charge transfer (ICT) of lysosome triggering dye (Fig. 5)^15^. Pyridine scaffolds got protonated at low pH and thus intramolecular charge transfer occurs, resulting in fluorescence spectrum intensity increases^13^.

### In vitro co-localization experiment

#### Cell viability assay

**Figure 15:**
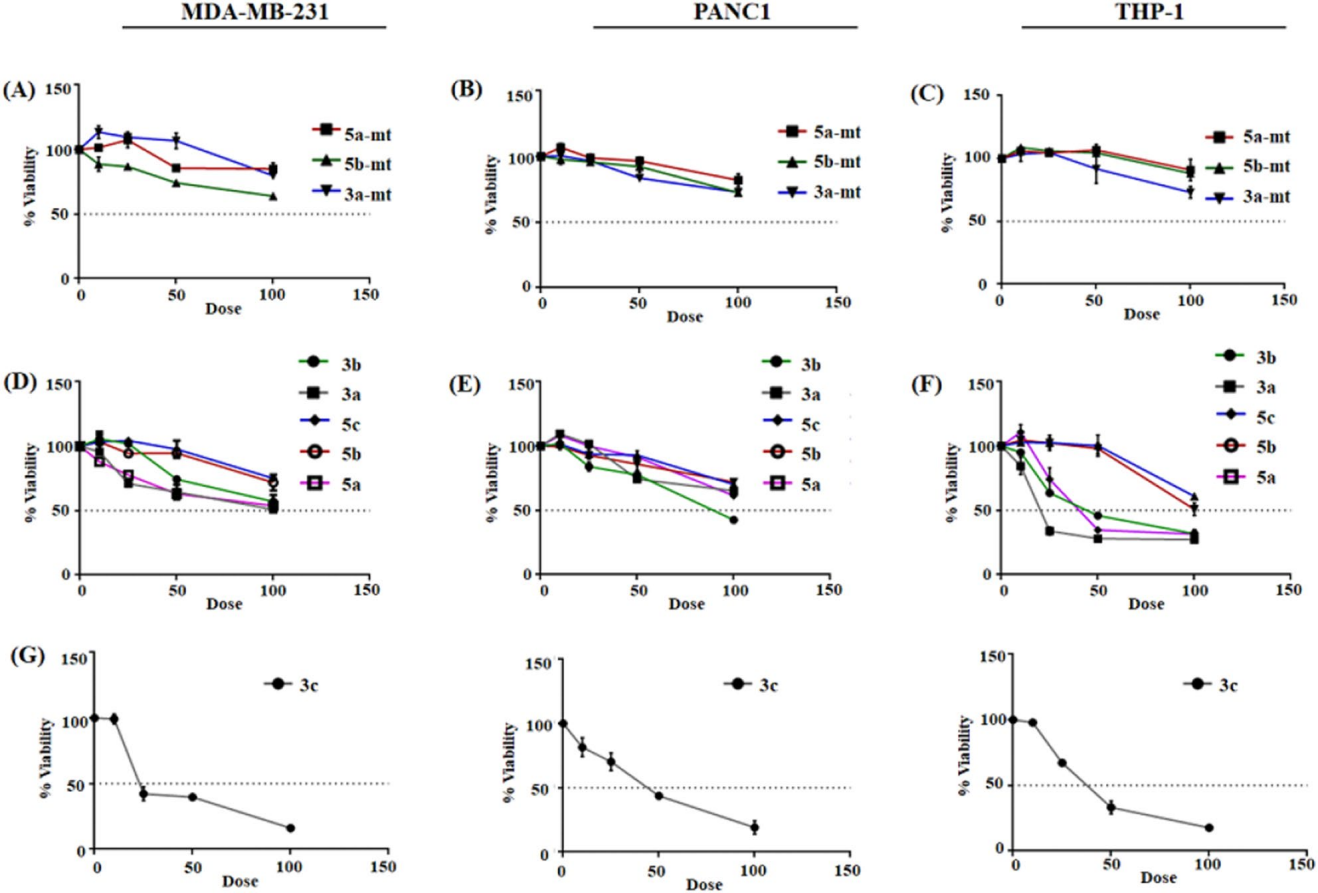
Cellular viability was estimated from MTT assay after 24-h incubation with varying concentrations of synthesized compounds (in μM). Cell viability data has been estimated from at least three independent experiments.

The cytotoxicity of the oxazole derivatives was first determined in different cell lines before evaluating the in vitro imaging capability. Three representative cell lines: a human breast cancer cell line: MDA-MB-231, human pancreatic cancer cell line (PANC-1) and a human leukemia monocytic cell line (THP-1) was selected for the study. Two of these are adherent cell lines (MDA-MB-231, PANC-1) and one is a suspension cell line (THP-1). These cell lines are commonly used in research and may give us an idea about how the dyes may function in vivo. The 3-(4,5-dimethylthiazol-2-yl)-2,5-diphenyl tetrazolium bromide (MTT) assay reveals no obvious cytotoxicity of oxazole derivatives at concentrations as high as 100 μM following 24 h incubation at 37 °C, 5% CO_2_ (Fig. 15). Recently, few reports reveal that, amino group containing lysosomal probes can usually cause an increase in cell mortality upon long-term incubation due to the alkaline effects from their amino groups which change the acidic environment within the lysosomes. Henceforth, the low cytotoxicity of these oxazole derivatives will make it more advantageous towards the application of long-term imaging for both mitochondria and lysosome. The compound **3c** however was found to be exceptional and affected cell viability.

### Cytoplasm, lysosome and mitochondria-specific cell staining

**Figure 16:**
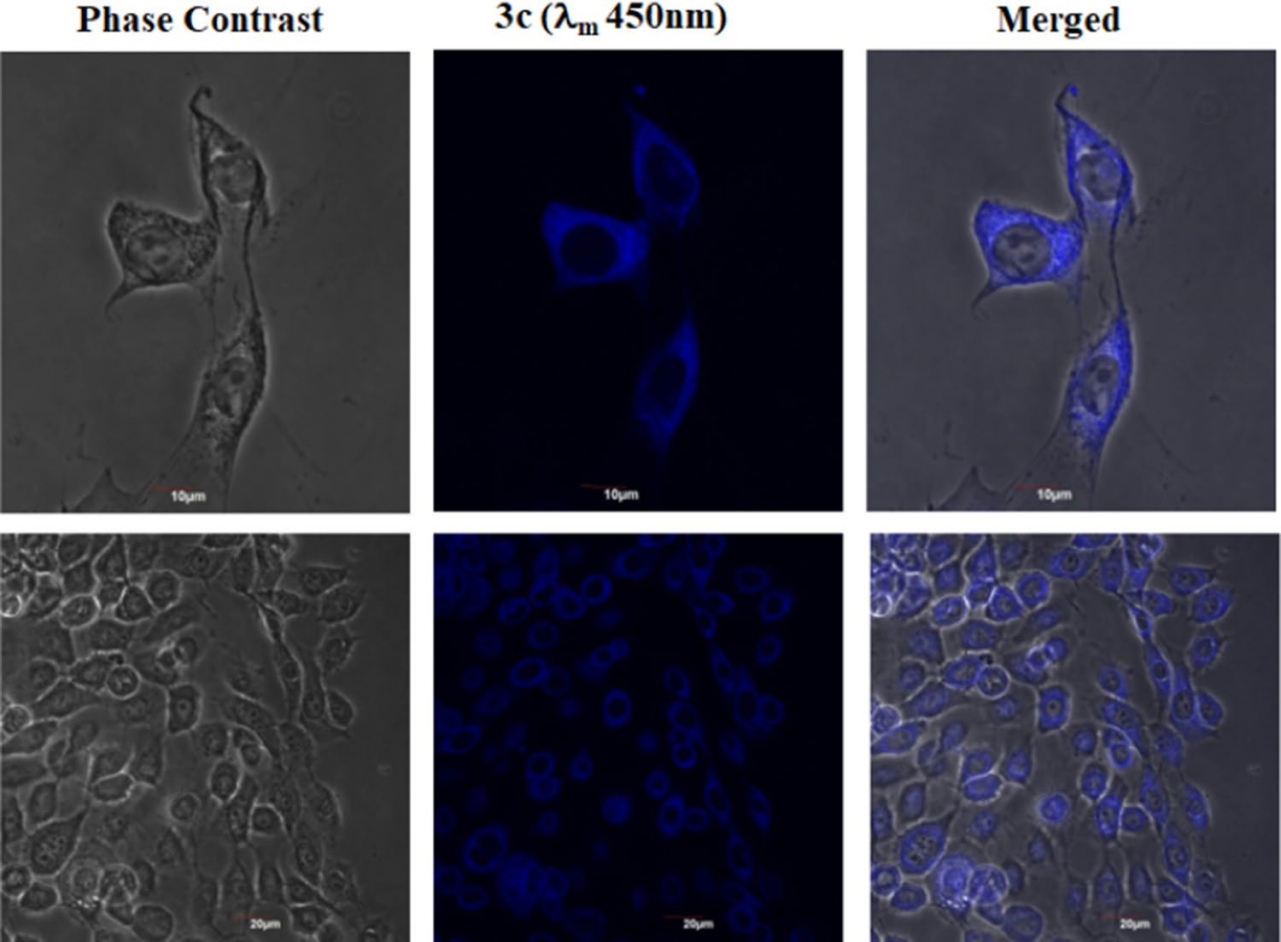
MDA-MB-231 cells were stained with **3c** (10 μM) for 30 min at 37 °C, fixed and observed by confocal microscopy. Cells were also imaged at higher magnification to check for the localization of the dye in the cytosol. A zoomed in view of cells have also been provided.

We initially checked if the synthesized compound **3c** was able to enter into cells by crossing the selectively permeable cell membrane. This synthesized compound showed good fluorescence property but did not have any targeting groups. MDA-MB-231 cells were incubated with compound **3c** and visualized via confocal laser scanning microscopy (CLSM). The compound **3c** was observed to selectively accumulate inside the cell cytoplasm in MDA-MB-231 cells (Fig. 16) which indicated that these small molecules can enter inside cells.

**Figure 17:**
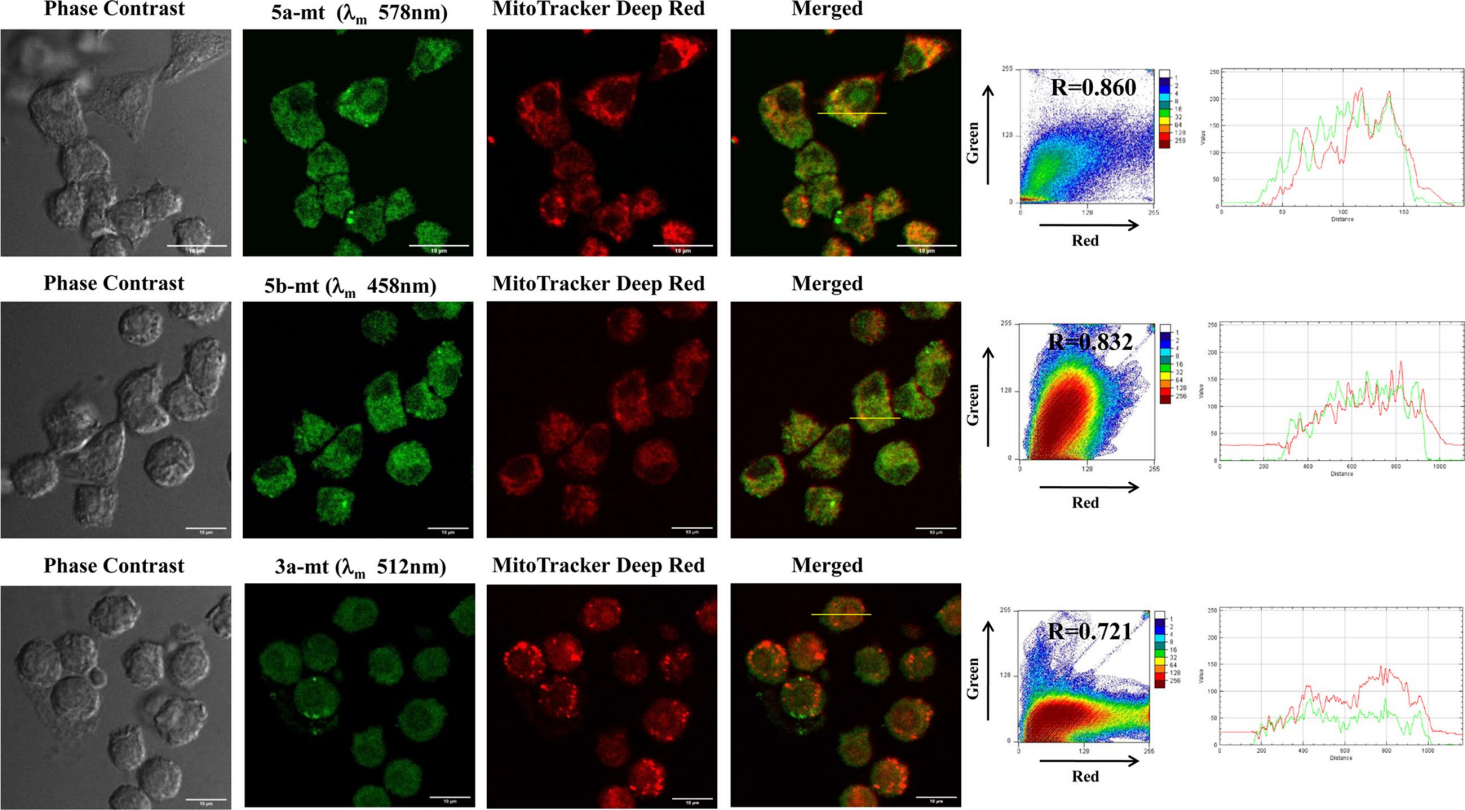
MDA-MB-231 cells were stained with **5a-mt**, **5b-mt **and **3a-mt** compounds (10 μM) along with Mitotracker Deep Red (50 nM). Cells were observed via confocal microscopy. Fluorescent signals of synthesized compounds are colored green for easy observation. Colocalization between the synthesized molecules (green channel) and Mitotracker Deep Red dye (red channel) was quantified from Pearson’s correlation coefficients. Two-dimensional (2D) histograms, intensity profiles of RGB channels along selected linear region of interest (yellow lines) have been shown.

**Figure 18:**
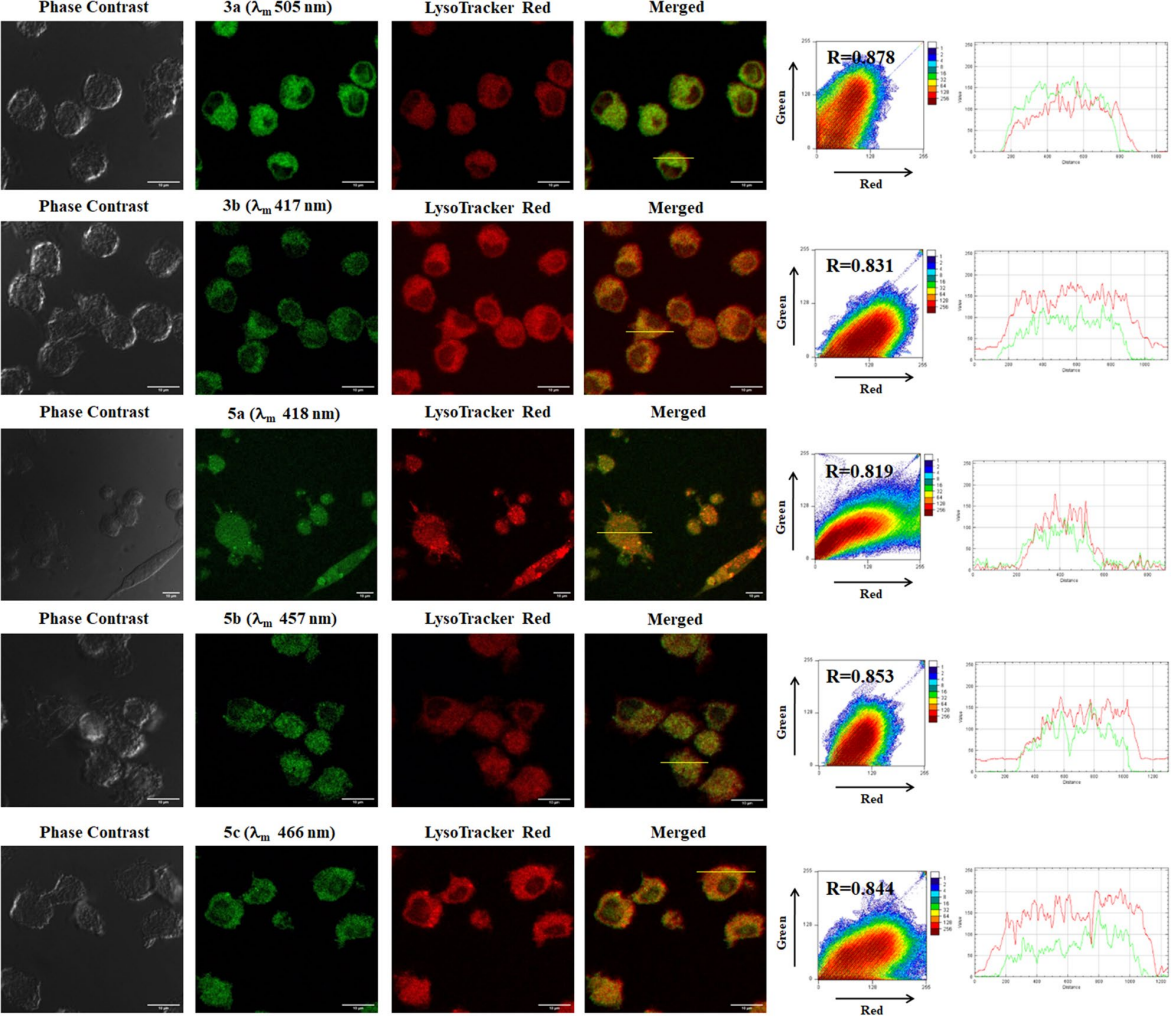
MDA-MB-231 cells were stained with **3a, 3b, 5a, 5b,** and **5c** (10 μM) respectively along with LysoTracker Red (75 nM) dye and visualized via confocal microscopy to observe cellular colocalization. Colocalization between the synthesized molecules (green channel) and LysoTracker Red (red channel) was quantified by calculating Pearson’s correlation coefficient. Two-dimensional (2D) histograms as well as intensity profiles of RGB channels along selected linear region of interest (yellow lines) are also shown.

Encouraged by this observation, we synthesized other compounds with organelle targeting groups. Pyridinium containing cationic scaffold **5a-mt, 5b-mt** and **3a-mt** was strategically designed such that these compounds may accumulate into mitochondria due to the electrostatic interaction between the cationic moiety and the negative mitochondrial membrane potential (MMP). For lysosome, the structure of **5a, 5b, 5c, 3a** and **3b** suggested that it would act as an acidotropic compound (a weak base that is membrane permeant in its unprotonated state) and should therefore accumulate in acidic organelle lysosome^11,15^. To confirm this speculation, localization of these synthesized molecules in cell lines were investigated by bioimaging via confocal laser scanning microscopy (Figs. 17 and 18). As these dyes did not show cytotoxicity in the MTT assay, 10 μM concentrations of dyes were used for in vitro staining.

Mitochondria membrane potential (MMP) is a key parameter for mitochondrial functions. This potential gradient arrives when mitochondria synthesize adenosine triphosphate (ATP) in its matrix through mitochondrial respiratory chain complex. ATP is the energy currency of the cell. So, it is extremely important to maintain mitochondrial membrane potential. Its depletion results in mitochondrial as well as cellular death^16^. The synthesized dyes were localized inside the mitochondria due to electrostatic interaction between negatively charged MMP and positively charged dye^14^.

For checking the mitochondrial localization of the synthetic oxazole derivatives **5a-mt, 5b-mt** and **3a-mt**, MDA-MB-231 cells were co-stained with both Mitotracker green and synthesized dyes (10 μM) (Fig. 17). The Pearson co-efficient of mitochondria staining dyes **5a-mt, 5b-mt** and **3a-mt** are 0.860, 0.832 and 0.721 respectively indicating that these probes can specifically accumulate inside the mitochondria (Fig. 17). Microscopy with the synthesized compounds also revealed that cellular morphology remained unaffected after incubation with 10 μM concentration of synthesized molecules indicating biocompatibility towards these probes. Localization and fluorescence of synthesized dyes were also unaffected by para-formaldehyde based fixation and processing.

To detect the lysosomal localization of the respective oxazole derivatives, MDA-MB-231 cells were co-stained with synthetic oxazole derivatives (10 μM) and Lysotracker DND-99, a commercial lysosome staining dye. The green punctated fluorescence of synthetic oxazole derivatives was observed inside cells, indicating the dyes are cell membrane permeable (Fig. 18). Colocalization of synthesized dyes and commercial lysotracker was analysed by estimating Pearson’s co-efficient. The Pearson’s correlation coefficient values of **3a, 3b, 5a, 5b** and **5c** were 0.878, 0.831, 0.819, 0.853 and 0.844 respectively, implying that these probes can selectively accumulate inside the lysosome.

## Conclusion

In this study a novel class of oxazole-based organelle targetable fluorescence probes (OTFPs) were synthesized using one-pot green synthetic approach from easily available starting materials. Different OTFPs can be easily tuned using this methodology simply by manipulating the probing groups. All these fluorophores were biocompatible and specifically localize inside the selective organelle like mitochondria, and lysosome with high Pearson’s correlation co-efficient. Moreover, by using the same methodology, cytoplasm stainer has also been developed which selectively and efficiently accumulate inside the cytoplasm only. Thus, by using the same organic synthetic methodology, different organelle targetable fluorophores can be easily synthesized and also can be further tuned accordingly. All the important information about these cellular organelles can be gathered by using these new versions of bio-probes.

## Experimental section

### General information

All reagents and solvents were purchased from Sigma Aldrich along with Thermo Fischer Scientific, Alfa Aesar and TCI Chemicals, were used without further purification. Open capillary tube was used for melting point determination. The poly-arylatedoxazoles were characterized by ^1^H, ^13^C NMR, ESI-mass, EI-mass spectroscopy. NMR spectra were recorded on a Bruker 600 MHz spectrometer instrument using CDCl_3_ or DMSO-d_6_ as solvent and tetramethylsilane (TMS) as an internal standard at room temperature. Chemical shifts are given in δ parts per million (ppm) relative to TMS, and the coupling constants *J* are given in Hertz. Copies of both ^1^H and ^13^C NMR spectroscopy can be found in the supporting information. High-resolution mass spectroscopes (HRMS) (m/z) were performed using EI techniques (JEOL-JMS 700 spectrometer). Bruker Kappa Apex **II** X-ray crystallography machine was used to solve the crystal structure.

### Cell lines and cell culture

The cell lines used in the study include—MDA-MB-231 (human breast cancer cell line), PANC-1 (human pancreatic cancer cell line) and THP-1 (human monocytic cell line) which were obtained from the National Cell Repository, National Centre for Cell Science, Pune, India. PANC-1, MDA-MB-231 cells were cultured in Iscove’s Modified Dulbecco’s Medium (IMDM) with 10% fetal calf serum (FCS) supplementation. THP-1 cells were cultured in Roswell Park Memorial Institute (RPMI) medium supplemented with 10% FCS. Cells were cultured at 37 °C, humidified condition with 5% CO_2_.

### Cell viability assay

Cellular toxicity from the compounds was determined by performing MTT assay. MDA-MB-231, PANC-1 and THP-1 cells (1 × 10^4^ cells/well) were seeded in 96-well plates. The synthesized compounds were added to the wells to various final concentrations (10, 25, 50 and 100 μM). Cells were incubated in presence of these molecules for 24 h at 37 °C. Following treatment, 3-(4,5-dimethylthiazol-2-yl)-2,5-diphenyl tetrazolium bromide(MTT) solution (100 μg/well, dissolved in medium) was added to each well of the 96-well plate, and further incubated at 37 °C for 3 h for formazan crystal formation. Medium was carefully removed from the wells, 200 µl of DMSO was added to dissolve the crystals and intensity of developing colour was measured by a plate reader at 550 nm. Experiments were performed in triplicate and the relative cell viability (%) was expressed as a percentage relative to the untreated control cells.

### Microscopy

MDA-MB-231 cells (1 × 10^5^) were seeded on sterilized poly-lysine coated cover slips for overnight attachment. Synthesized molecules designed to target mitochondria (**5a-mt, 5b-mt, 3a-mt**) were added to the culture medium at 10 μM concentration and incubated for 20 min at 37 °C. Simultaneously, commercially available mitochondria specific stainer Mitotracker Deep Red (50 nM) was also added and incubated for 15 min at 37 °C. On other hand, cells similarly treated with lysosome-directing compounds (**3a, 3b, 5a, 5b and 5c**), were incubated with Lysotracker Red (75 nM) for 20 min respectively. After dual staining, cells were washed twice with phosphate buffered saline (0.02 M, PBS pH 7.2) followed by 2% paraformaldehyde fixation. Cells were washed with PBS again and then mount on slides, sealed. Slides were observed through super resolution confocal microscope (Andor Spinning Disc Confocal microscope). Images were finally analyzed by ImageJ software (NIH) and degree of colocalization was estimated from Pearson’s correlation coefficient determined using JaCOP plugin.

### Spectroscopic measurements

All the solutions were prepared using spectroscopic grade solvent. Fluorescence experiments were performed using a Fluorescence Spectrophotometer F-7000 Hitachi, equipped with double monochromators in both excitation and emission. All the experiments carried out using high-quality quartz cuvettes at room temperature.

### General experimental procedure for the preparation of 2,4,5-trisubstituted oxazoles (3c, 5c)

To a mixture of benzil (**1a**) (0.50 mmol, 1 equiv.), and amine **(2c-d)** (0.50 mmol, 1 equiv) iodine (0.15 mmol, 0.3 equiv.) was added in H_2_O (3 ml) in open air and it was heated at 60 °C for 8 h. The completion of the reaction was monitored by checking TLC. The reaction mixture was then allowed to cool to room temperature and subsequently solid appeared. After that, the reaction mixture was treated with 10% aqueous Na_2_S_2_O_3_. The aqueous phase was then extracted with EtOAc and the organic phase was dried by anhydrous Na_2_SO_4_, filtered and concentrated under reduced pressure. The crude mixture was then purified by column chromatography (silica gel, 100–200 mesh) using a mixture of EtOAc and petroleum ether as eluent to afford the desired product **(3c, 5c).** The other oxazole derivatives (**3a, 3b, 5a, 5b**) were prepared and characterized using standard procedure^14^.

### General experimental procedure for the preparation of 3a-mt, 5a-mt, 5b-mt and 5c-mt

To a solution of **3a** (0.50 mmol, 1 equiv.) in toluene (3 ml), methyl iodide (1.0 mmol, 2equiv.) was added. The resulting solution was stirred at room temperature for two hours. The completion of the reaction was monitored by checking TLC. The reaction mixture was then evaporated by rotary evaporator and washed with diethyl ether. Then the washed compound was dried over rotary evaporator.

#### 4-(4-(2-chlorophenyl)-5-(3,4-dimethoxyphenyl)oxazol-2-yl)-1-methylpyridin-1-ium (5a-mt)

Yellow solid; Yield (**5a-mt** 99%); ^1^H NMR (600 MHz, DMSO-d6): δ 9.09 (d, *J* = 6.6 Hz, 2H), 8.70 (d, *J* = 6.0 Hz, 2H), 7.70 (d, *J* = 7.8 Hz, 1H), 7.64 (d, *J* = 7.2 Hz, 1H), 7.60 (t, *J* = 7.2 Hz, 1H), 7.55 (t, *J* = 7.2 Hz, 1H), 7.09 (m, 2H), 7.03 (m, 1H), 4.37 (s, 3H), 3.77 (s, 3H), 3.63 (s, 3H); ^13^C NMR (150 MHz, DMSO-d6): δ 154.53, 150.86, 150.58, 149.33, 146.94, 139.48, 135.01, 133.59, 132.77, 132.00, 131.13, 130.49, 128.47, 123.45, 119.40, 119.36, 112.57, 109.13, 56.11, 55.81, 48.23; (ESI +-HRMS) calculated: C_23_H_20_N_2_O_3_Cl (M+): 407.1162, Found: 407.1165.

#### 3-(4-(2-chlorophenyl)-5-(3,4-dimethoxyphenyl)oxazol-2-yl)-1-methylpyridin-1-ium (5b-mt)

Yellow solid; Yield (**5b-mt** 98%); ^1^H NMR (600 MHz, DMSO-d6): δ 9.76 (s, 1H), 9.17–9.16 (m, 1H), 9.07 (s, 1H), 8.28 (s, 1H), 7.69 (s, 1H), 7.62–7.55 (m, 3H), 7.10–7.04 (m, 3H), 4.45 (s, 3H), 3.77 (s, 3H), 3.61 (s, 3H); ^13^C NMR (150 MHz, DMSO-d6): δ162.64, 157.85, 153.81, 150.45, 148.99, 147.74, 147.33, 146.64, 146.25, 145.49, 143.85, 141.17, 130.40, 128.62, 128.35, 126.60, 119.91, 118.89, 112.56, 108.99, 65.34, 56.04, 53.20; (ESI+-HRMS) calculated: C_23_H_20_N_2_O_3_Cl (M+): 407.1162, Found: 407.1164.

#### 4-(4,5-diphenyloxazol-2-yl)-1-methylpyridin-1-ium (3a-mt)

Yellow solid; Yield (**3a-mt** 99%); ^1^H NMR (600 MHz, MeOD): δ 9.03 (d, *J* = 6.6 Hz, 2H), 8.67 (d, *J* = 7.2 Hz, 2H), 7.78–7.76 (m, 2H), 7.73–7.71 (m, 2H), 7.51–7.47 (m, 6H), 4.47 (s, 3H); ^13^C NMR (150 MHz, DMSO-d6): δ 155.46, 149.20, 147.02, 139.69, 138.70, 131.33, 130.68, 129.64, 129.44, 128.32, 127.51, 127.37, 125.48, 123.62, 48.28; (ESI +-HRMS) calculated: C_21_H_17_N_2_O (M^+^): 313.1335, Found: 313.1333.

#### 4-(2-chlorophenyl)-5-(3,4-dimethoxyphenyl)-2-(pyridin-2-yl)oxazole (5c)

Pale yellow solid; Yield (**5c** 76%); ^1^H NMR (600 MHz, CDCl_3_): δ 9.35 (s, 1H), 8.66 (s, 1H), 8.40 (d, *J* = 7.8 Hz, 1H), 7.55–7.51 (m, 2H), 7.42–7.37 (m, 3H), 7.11 (d, *J* = 7.8 Hz, 1H), 6.91 (s, 1H), 6.83 (d, *J* = 8.4 Hz, 1H), 3.86 (s, 3H), 3.66 (s, 3H); ^13^C NMR (150 MHz, CDCl_3_): δ 166.85, 158.17, 149.98, 149.46, 148.28, 146.02, 137.28, 134.42, 133.22, 132.28, 130.14, 127.06, 126.45, 124.50, 122.42, 120.85, 118.75, 111.06, 108.62, 55.83, 55.59; (ESI +-HRMS) calculated: C_22_H_18_N_2_O_3_Cl (M+H^+^): 393.1006, Found: 393.0999.

#### 4-(4,5-diphenyloxazol-2-yl)benzaldehyde (3c)

White solid; Yield (**3c** 76%); ^1^H NMR (600 MHz, DMSO-d6): δ 10.09 (s, 1H), 8.32 (d, *J* = 8.4 Hz, 2H), 8.09 (d, *J* = 7.8 Hz, 2H), 7.70 (d, *J* = 7.8 Hz, 2H), 7.67 (d, *J* = 7.8 Hz, 2H), 7.50–7.43 (m, 6H); ^13^C NMR (150 MHz, DMSO-d6): δ 146.69, 137.13, 132.06, 131.67, 130.75, 129.86, 129.53, 129.28, 129.14, 128.31, 128.17, 127.13, 127.07; (ESI +-HRMS) calculated: C_22_H_15_NO_2_ (M+H+): 326.1176, Found: 326.1174.

## Supplementary Information

Supplementary Information.

## Data Availability

All data generated or analysed during this study are included in this published article [and its supplementary information files].
