# Supplementary material for: Cell-imaging studies of highly substituted oxazole derivatives as organelle targeting fluorophores (OTFPs)
Source: Sci Rep. 2022 Oct 3;12:16555. doi: 10.1038/s41598-022-20112-y (PMC9530160; doi:10.1038/s41598-022-20112-y)
Supplement: Supplementary file 1 — Supplementary Information. [file 41598_2022_20112_MOESM1_ESM.doc]

**Supporting Information**

**Cell-Imaging Studies of Highly Substituted Oxazole Derivatives as Organelle Targeting Fluorophores (OTFPs)**

Saswati Adhikary,^a^ Kaustuv Mukherjee,^c^ and Biswadip Banerji*^,a,b^

*^a^Organic and Medicinal Chemistry Division, Indian Institute of Chemical Biology (CSIR-IICB), 4 Raja S. C. Mullick Road, Kolkata, 700032, India.*

*^b^Academy of Scientific and Innovative Research (AcSIR), Ghaziabad-201002, India. Email: biswadip.banerji@gmail.com, biswadip@iicb.res.in; Fax: +91 33 24735197, +91 33 24723967; Tel : + 91 33 24995709.*

*^c^Cancer Biology & Inflammatory Disorder Division, Indian Institute of Chemical Biology (CSIR-IICB), 4 Raja S. C. Mullick Road, Kolkata, 700032, India.*

**Contents:**

**Table of contents: Page No.**

**1. ^1^H and ^13^C NMR spectral data S3-S12**

**2. ORTEP diagram of product 5a S13**

**3. X-ray crystallographic information of product 5a S13-S14**

**4. Photostability of fluorophore S14**

**5. References S15**

**Figure S1: ^1^H-NMR spectrum of 4-(4-(2-chlorophenyl)-5-(3,4-dimethoxyphenyl)oxazol-2-yl)-1-methylpyridin-1-ium (5a-mt) in DMSO-d_6_.**

**
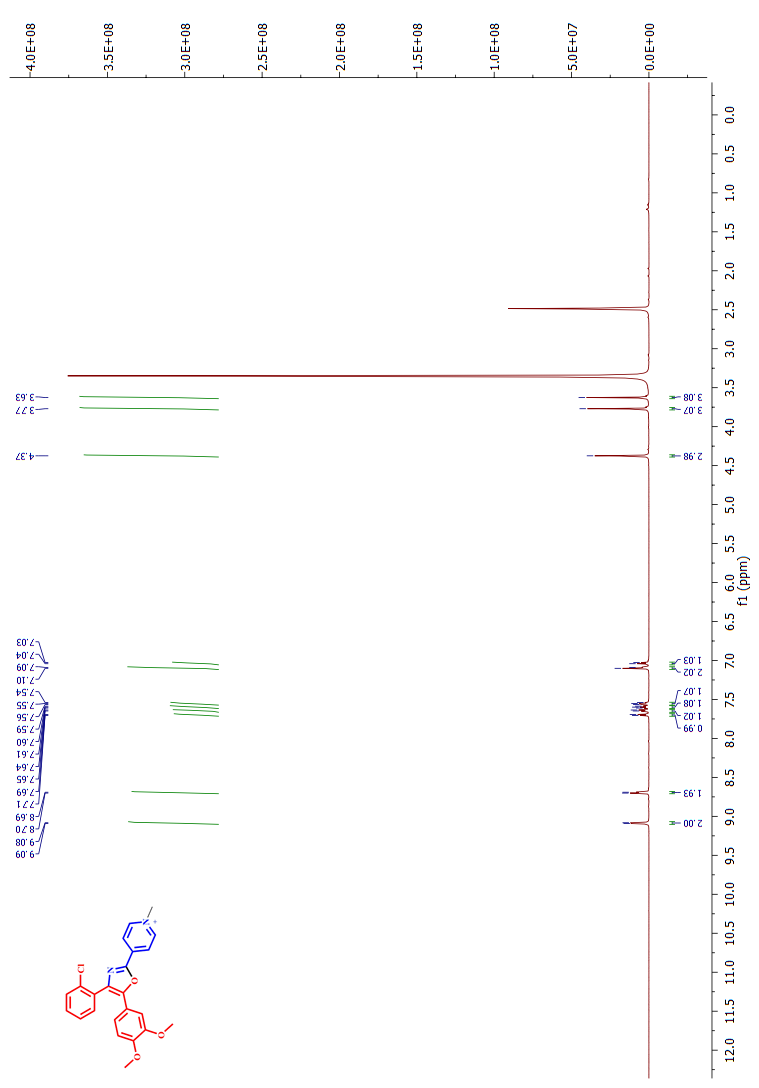
**

**Figure S2: ^13^C-NMR spectrum of 4-(4-(2-chlorophenyl)-5-(3,4-dimethoxyphenyl)oxazol-2-yl)-1-methylpyridin-1-ium (5a-mt) in DMSO-d_6_.**

**
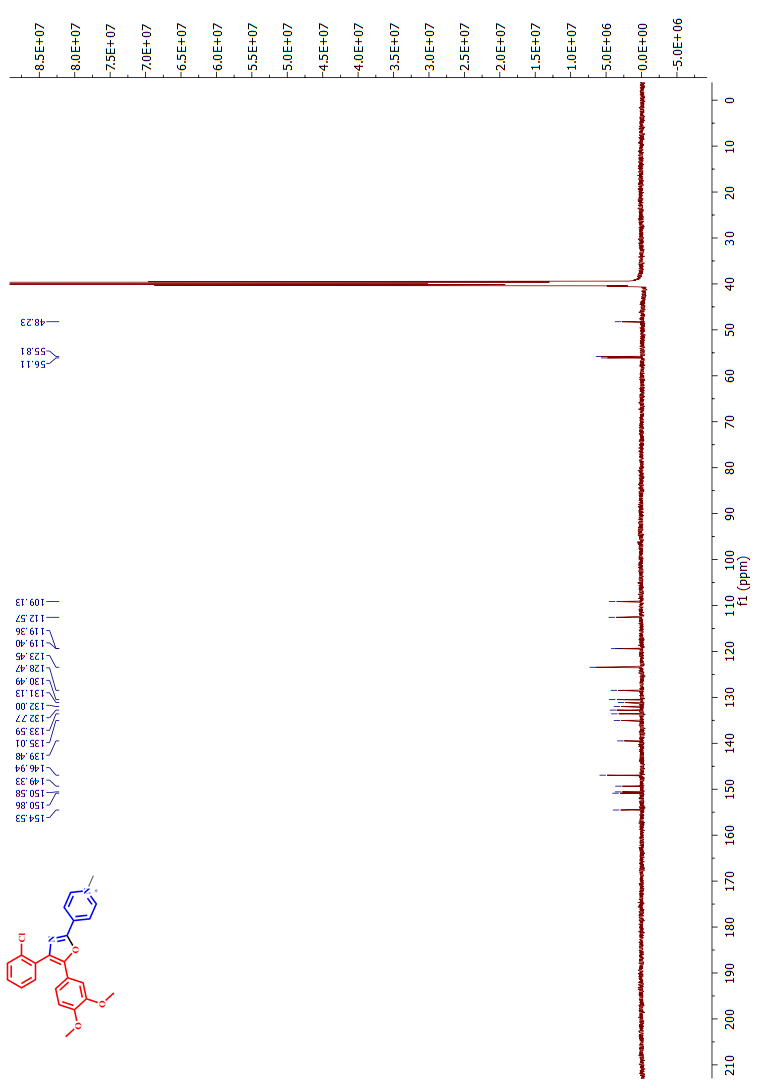
**

**Figure S3: ^1^H-NMR spectrum of 3-(4-(2-chlorophenyl)-5-(3,4-dimethoxyphenyl)oxazol-2-yl)-1-methylpyridin-1-ium (5b-mt) in DMSO-d_6_.**

**
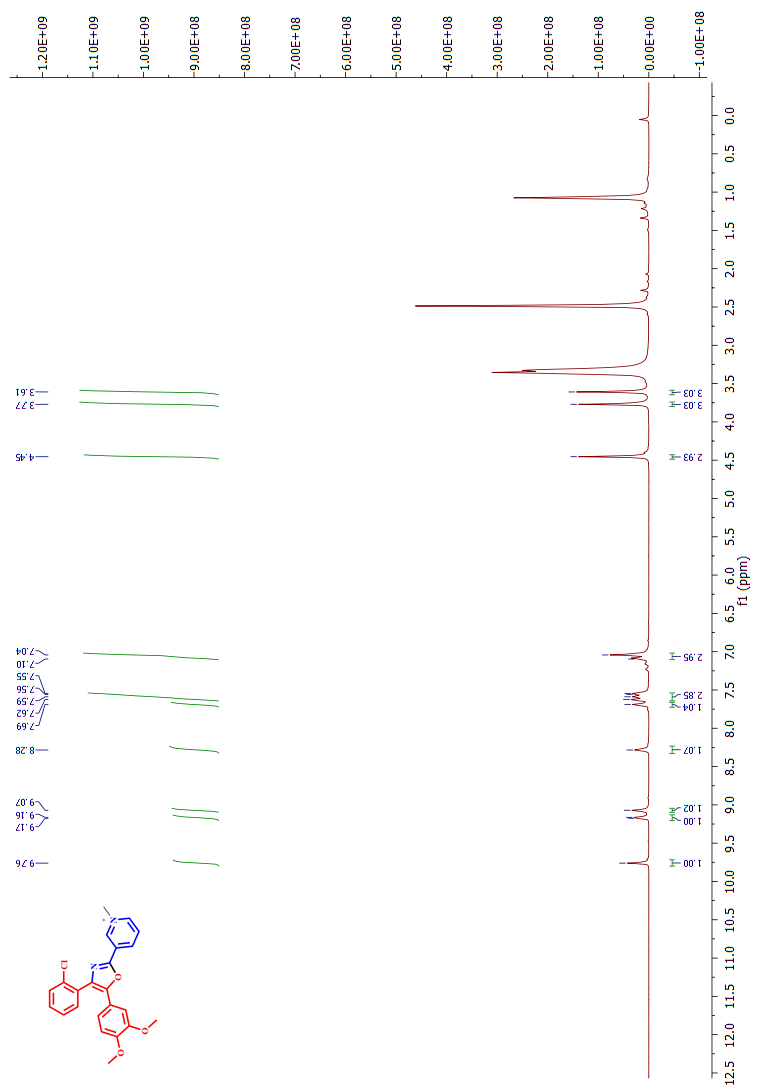
**

**Figure S4: ^13^C-NMR spectrum of 3-(4-(2-chlorophenyl)-5-(3,4-dimethoxyphenyl)oxazol-2-yl)-1-methylpyridin-1-ium (5b-mt) in DMSO-d_6_.**

**
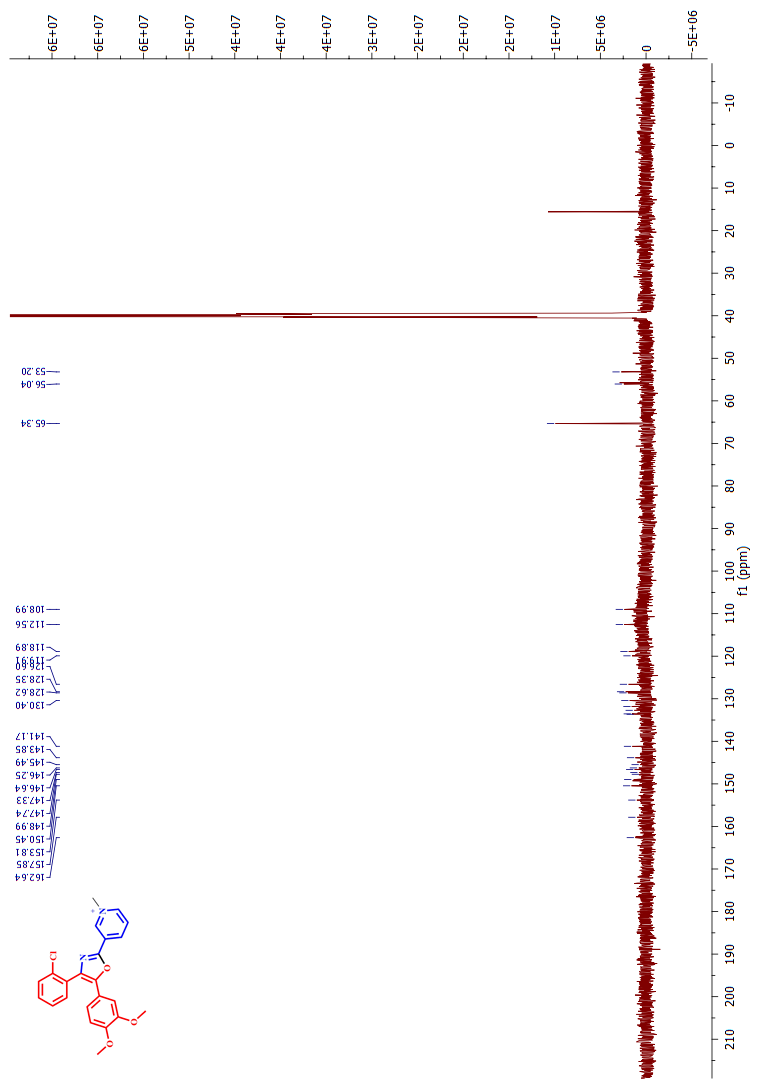
**

**Figure S5: ^1^H-NMR spectrum of 4-(4,5-diphenyloxazol-2-yl)-1-methylpyridin-1-ium (3a-mt) in MeOD.**

**
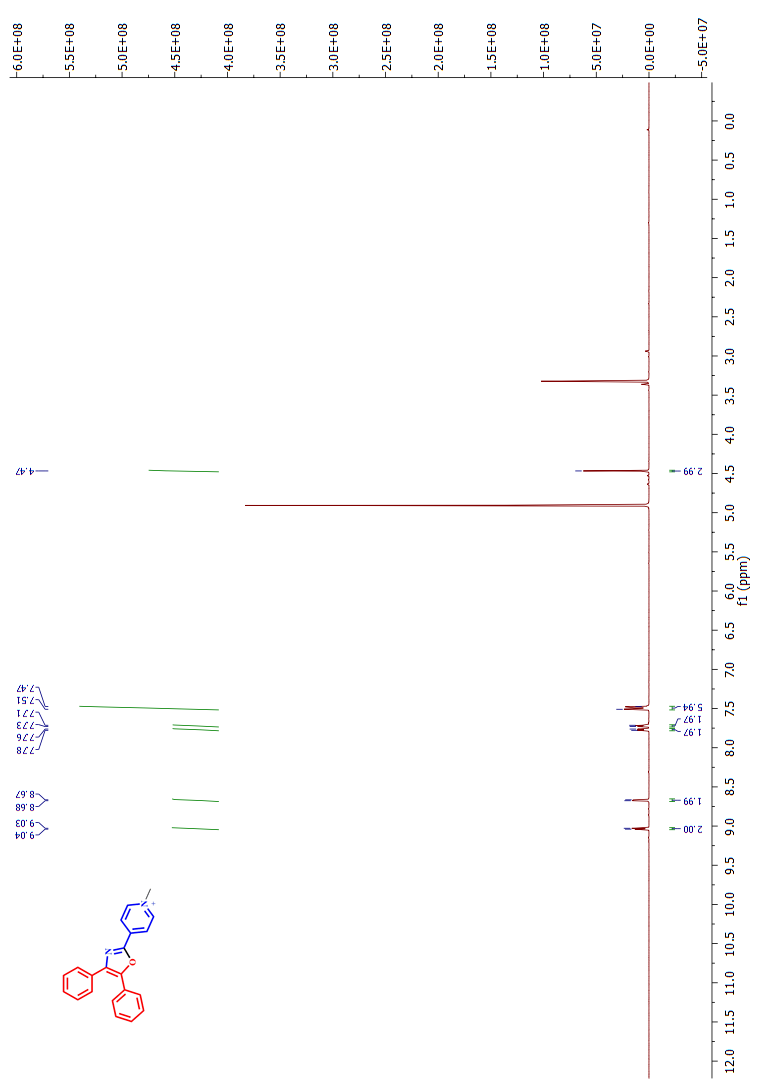
**

**Figure S6: ^13^C-NMR spectrum of 4-(4,5-diphenyloxazol-2-yl)-1-methylpyridin-1-ium (3a-mt) in DMSO-d_6_.**


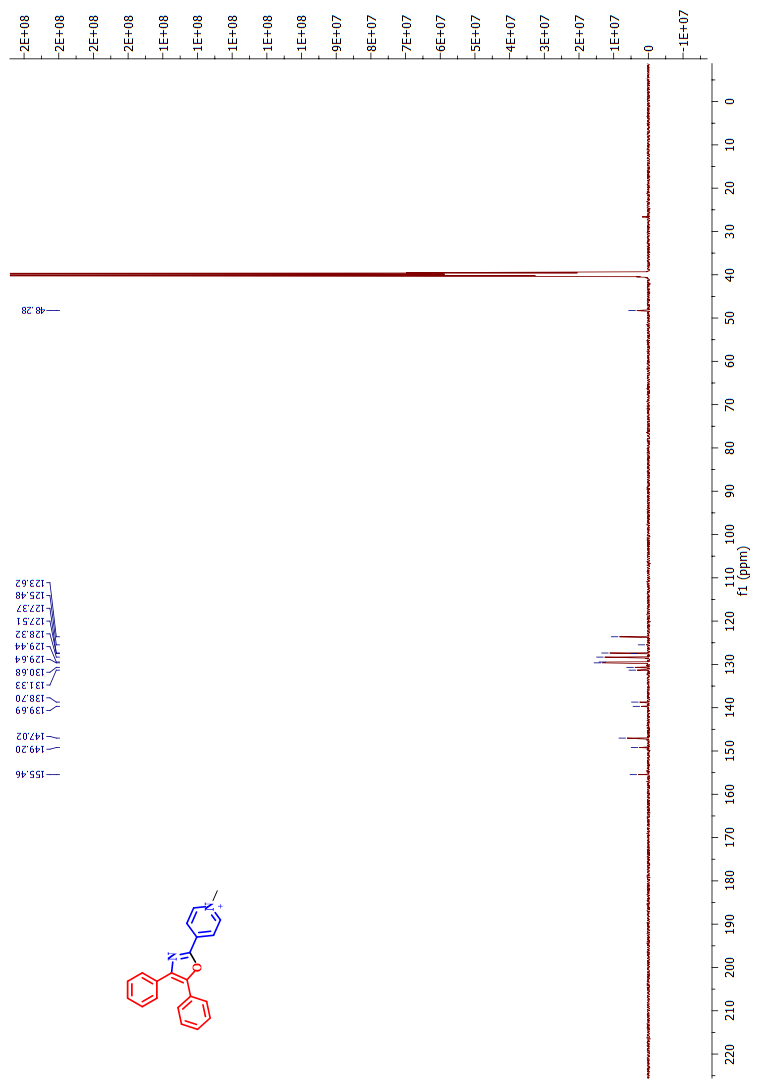


**Figure S7: ^1^H-NMR spectrum of 4-(2-chlorophenyl)-5-(3,4-dimethoxyphenyl)-2-(pyridin-2-yl)oxazole (5c) in CDCl_3_.**


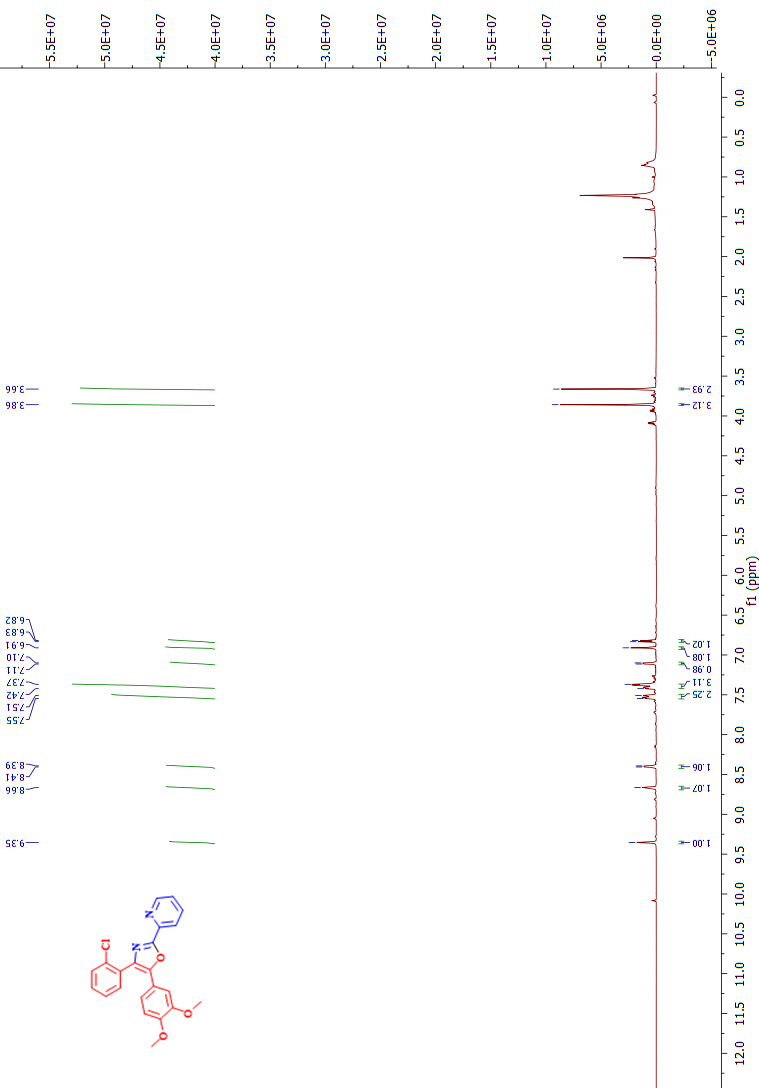


**Figure S8: ^13^C-NMR spectrum of 4-(2-chlorophenyl)-5-(3,4-dimethoxyphenyl)-2-(pyridin-2-yl)oxazole (5c) in CDCl_3_.**


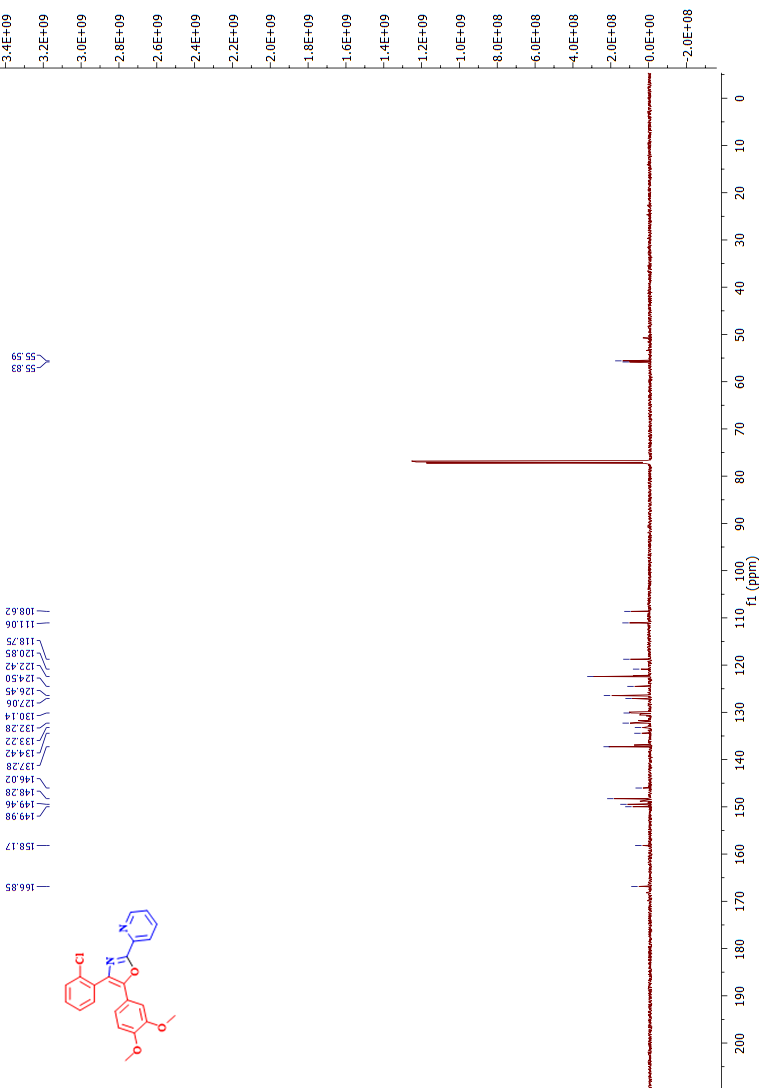


**Figure S9: ^1^H-NMR spectrum of 4-(4,5-diphenyloxazol-2-yl)benzaldehyde (3c) in DMSO-d_6_.**


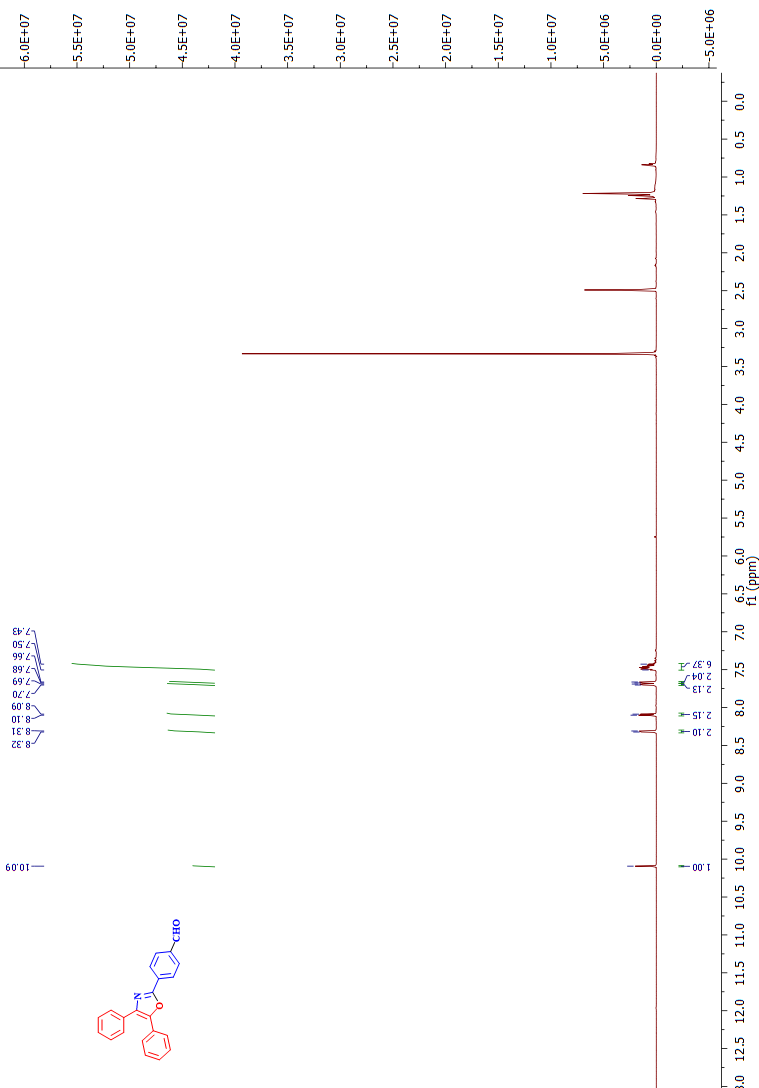


**Figure S10: ^13^C-NMR spectrum of 4-(4,5-diphenyloxazol-2-yl)benzaldehyde (3c) in DMSO- d_6._**


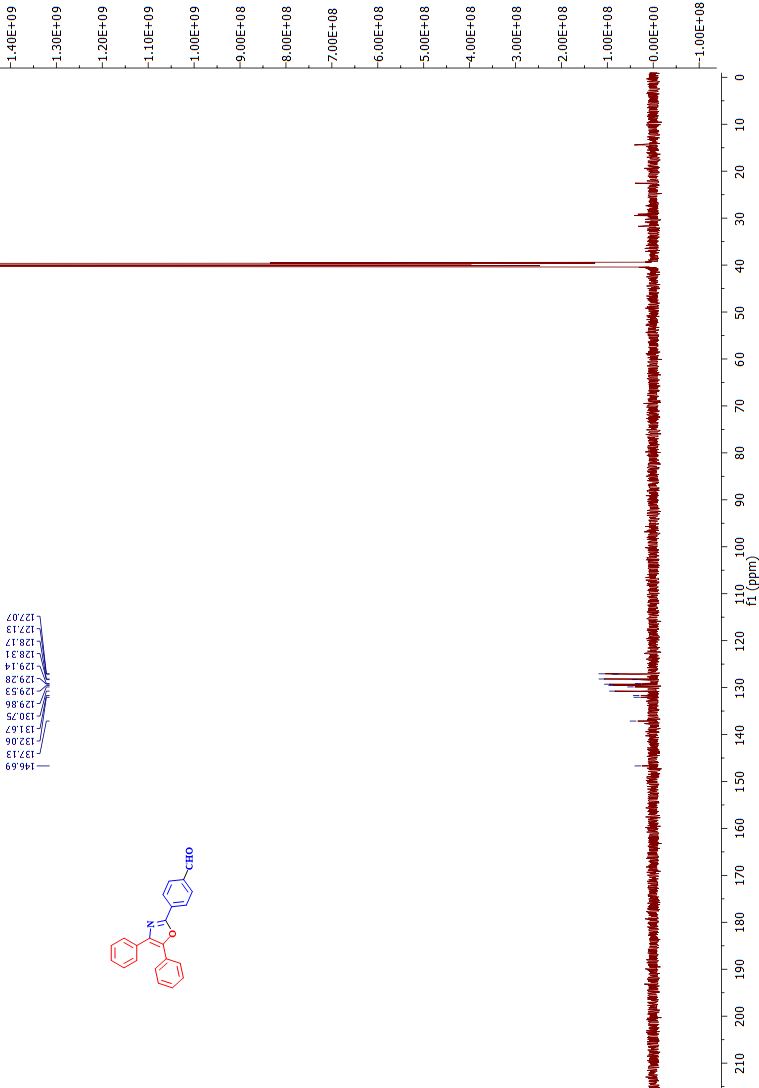


X-ray Diffraction Analysis of Compound **5a** :

**
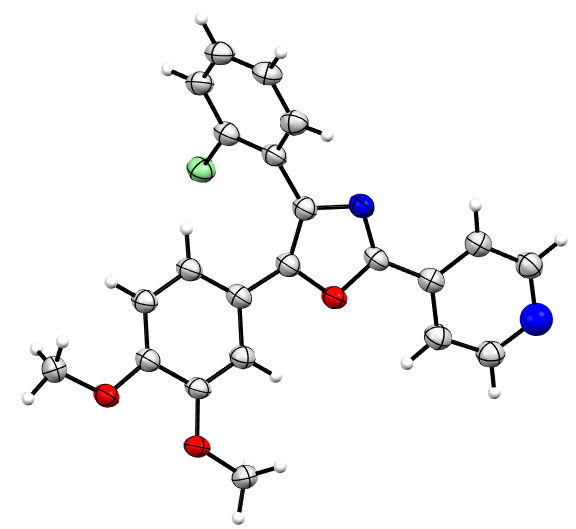
**

**Figure S11.** ORTEP diagram (thermal ellipsoid) of product **5a** (drawn at 50% probability

level). Colour code: nitrogen in blue and carbon in grey, oxygen in red, chlorine in green, hydrogen in white.

**X-Ray Crystallographic Information of 5a:**

The Single crystal of highly substitutes oxazole derivative **5a** was developed by slow evaporation of hexane and dichloromethane at room temperature. With the MoKα radiation (λ = 1.54178Å) at 100 K, the diffraction data of compound **5a** was measured. By using the SHELXS-97 program, the single crystal structure of oxazole derivative **5a** was solved.^1^ Refinements were carried out by full matrix least-squares process against F using SHELXL-97.2 and the non-hydrogen atoms were refined with anisotropic thermal parameters.^2^ All the hydrogen atoms in the crystal structure (**5a**) were included in geometric positions. All the essential crystal data of the derivative **5a** is given below:

| **Table S1**: Important crystal data of product **5a.** | |
| --- | --- |
| Empirical Formula | C_22_ H_17_ Cl N_2_ O_3_ |
| Formula weight | 392.82 |
| Temperature | 100 K |
| Wave length | 1.54178 |
| Crystal system | monoclinic |
| Space group | C 1 2/c 1 |
| Unit cell dimensions | a = 35.682(5) Å; α = 90 ^o^  b = 4.9679(8) Å; β = 92.647(8) ^o^  c = 20.475(4) Å; γ = 90 ^o^ |
| Volume | 3625.7(10) Å^3^ |
| Z | 8 |
| Density (calculated) | 1.439 g /cm^3^ |
| Absorption coefficient (Mu) | 2.093 mm-1 |
| F(000) | 1632.0 |
| Theta range for data collection | 2.479^o^ to 69.008^o^ |
| Index ranges | -42 ≤ h ≤ 42, -5 ≤ k ≤ 5, -24 ≤ l ≤ 24 |
| Reflection collected | 60156 |
| Absorption correction | Multi-scan |
| Data/restraints/parameters | 3167/0/256 |

**The crystal data of product 5a was deposited at the Cambridge Crystallographic Data**

**Centre. The CCDC reference number is 2160453.**

**Photostability of fluorophore:**

**
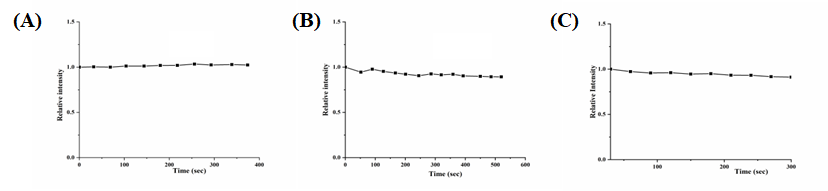
**

(A) Photostability of **5a-mt** in 10 μM buffer solution of pH 8.0, (B) Photostability of in 10 μM buffer solution of pH 8.0, (C) Photostability of **3a-mt** in 10 μM buffer solution of pH 8.0. **5b-mt**


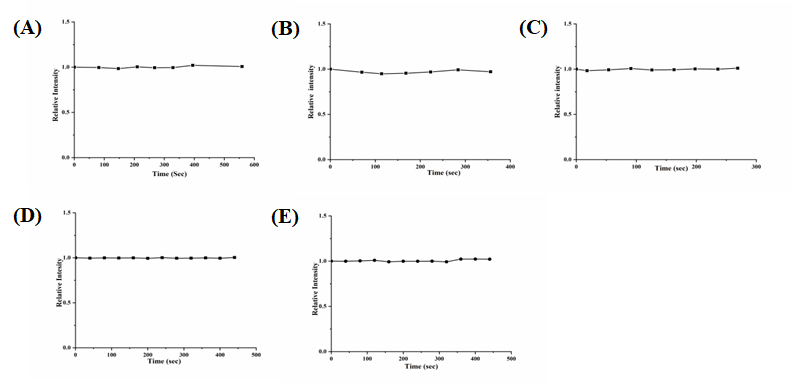


(A) Photostability of **5a** in 10 μM buffer solution of pH 4.5, (B) Photostability of **5b** in 10 μM buffer solution of pH 4.5, (C) Photostability of **5c** in 10 μM buffer solution of pH 4.5, (D) Photostability of **3a** in 10 μM buffer solution of pH 4.5, (E) Photostability of **3b** in 10 μM buffer solution of pH 4.5.

**4. References:**

1. Sheldrick, G. M.; Phase Annealing in SHELX-90: Direct Methods for Larger Structures.

*Acta Cryst.* **1990**, *A46,* 467-473.

2. Sheldrick, G. M. *SHELXL-97, Program for Crystal Structure Refinement;* Universität Göttingen: Göttingen, Germany, 1997.
